# Statistical analysis and first-passage-time applications of a lognormal diffusion process with multi-sigmoidal logistic mean

**DOI:** 10.1007/s00362-022-01349-1

**Published:** 2022-08-27

**Authors:** Antonio Di Crescenzo, Paola Paraggio, Patricia Román-Román, Francisco Torres-Ruiz

**Affiliations:** 1 Dipartimento di Matematica, Università degli Studi di Salerno, Via Giovanni Paolo II n. 132, 84084 Fisciano, SA Italy; 2 Department of Statistics and Operations Research, Faculty of Sciences, University of Granada, 18071 Granada, Spain; 3 Institute of Mathematics of the University of Granada (IMAG), Calle Ventanilla, 11, 18001 Granada, Spain

**Keywords:** Lognormal diffusion process, Multi-sigmoidal growth, Maximum likelihood estimation, Asymptotic distribution, First-passage-time, First-passage-time location function, 62M05, 60J70

## Abstract

We consider a lognormal diffusion process having a multisigmoidal logistic mean, useful to model the evolution of a population which reaches the maximum level of the growth after many stages. Referring to the problem of statistical inference, two procedures to find the maximum likelihood estimates of the unknown parameters are described. One is based on the resolution of the system of the critical points of the likelihood function, and the other is on the maximization of the likelihood function with the simulated annealing algorithm. A simulation study to validate the described strategies for finding the estimates is also presented, with a real application to epidemiological data. Special attention is also devoted to the first-passage-time problem of the considered diffusion process through a fixed boundary.

## Introduction

Growth curves with sigmoidal behavior are widely used in several applied fields including biology (see, for instance, Brauer and Castillo-Chavez (2012)), software reliability (cf. Erto et al. (2020), Inoue and Yamada (2013)) and economics (see, for example, Smirnov and Wang (2020)). During the times different kinds of sigmoidal curves have been introduced such as logistic, Gompertz, Korf, Bertalanffy, etc. Many efforts have been made essentially for two main purposes: (i) unification of classical models (see Chakraborty et al. (2019)), and (ii) generalizations of growth curves (see, for example, Asadi et al. (2020), Di Crescenzo and Spina (2016) and Romero et al. (2016)).

The differential equations which drive the growth of the aforementioned deterministic models are very useful to describe population dynamics. However, in order to make them more realistic, it is necessary to introduce a noise term in the equation. In this way, the differential equations are replaced by stochastic ones. Most of the times, the analysis of the resulting stochastic equation is quite complex, and the transition probability density of the resulting diffusion process cannot be determined (for example, see Campillo et al. (2018), in which the authors propose, for this reason, a new approach to find the maximum likelihood estimates). Models based on diffusion processes are commonly used in various fields of applications, for example plant dynamics (cf. Rupšys et al. (2020), where a hybrid growth is based on Gompertz and Vasicek models), resources consumption (for instance, Nafidi et al. (2019) use the Brennan-Schwartz process to model electricity consumption in Morocco) or particular fish species growth (cf. a stochastic version of the open-ended logistic model considered in Yoshioka et al. (2019)).

In a recent paper, Di Crescenzo et al. (2021) focus on the generalization of the classical logistic growth model introducing more than one inflection point. To this end, firstly, two different birth-death processes, one with linear birth and death rates and the other with quadratic rates, were considered. Then, a diffusive approximation was performed leading to a non-homogeneous lognormal diffusion process with mean of multi-sigmoidal logistic type. Attention was also given to the description of its main features of interest in applied contexts. For instance, the mean of the process is a generalized version of the classical logistic function (see, for instance, Di Crescenzo and Paraggio (2019)) with more than one inflection point. The transition probability density of the process has been obtained explicitly and has been applied to plant dynamics.

Starting from the theoretical results of the previous works, in the present paper we approach the problem of the inference of the stochastic model. This is done by means of the maximum likelihood method, thanks to the availability in closed form of the likelihood function. We also address the treatment of some collateral problems that emerge in the development carried out, such as: (i) obtaining initial solutions to solve the system of likelihood equations, and (ii) bounding the parametric space for addressing the estimation by metaheuristic procedures. All development is supported by simulation examples. Subsequently, in order to provide an example of application to real phenomena, we adopt the proposed model to describe the behavior of the data on the evolution of COVID-19 in different European countries during the two first waves of infection. Indeed, some of the main features of the diffusion process, such as the mean, the mode and the quantiles, may be used for prediction purposes and they are expressed as a function of the parameters of the process.

The problem of parameters estimation has been considered in several papers, for instance in Shimizu and Iwase (1987) and in Tanaka (1987). See also the more recent works of Garcia (2019), in which the author converts the maximization of the likelihood function into an equivalent problem regarding the minimization of a square error, and of Ramos-Ábalos et al. (2020) where maximum likelihood estimates of the parameters of the powers of the homogeneous Gompertz diffusion process are obtained.

Two different strategies to obtain the maximum likelihood estimates of the parameters are introduced. The first is based on the solution of the system of the critical points of the likelihood function, and the other stems from a meta-heuristic optimization method (simulated annealing) to maximize the likelihood function.

This is the outline of the content of the paper. In Sect. 2, the most relevant characteristics of the deterministic and the corresponding stochastic model are recalled. Then, the problem of finding the maximum likelihood estimates of the involved parameters is described in Sect. 3. In several contexts of population dynamics, it may be relevant to know how long the population spends below a certain control threshold. For this reason the first-passage-time (FPT) problem is also addressed. More precisely, in Sect. 4, the R-package fptdApprox (see Román-Román et al. (2020)) is used to determine the approximated FPT density of the lognormal diffusion process through a constant boundary. With the purpose of validating the described procedures for finding the maximum likelihood estimates, a simulation study is presented in Sect. 5. Finally, in Sect. 6 we propose an application of the model to real data concerning the COVID-19 infections in France, Italy, Spain and United Kingdom.

## The multisigmoidal logistic model and the corresponding diffusion process

Consider the classical logistic equation

$$\begin{aligned} \frac{d}{dt}l(t)= rl(t)\left[ 1-\frac{\eta }{C}l(t)\right] , \qquad t\ge t_0, \end{aligned}$$

with $r, \eta , C>0$. If the intrinsic growth rate *r* is replaced by a polynomial *P*(*t*), then the solution of this equation, with the initial condition $l(t_0)=l_0$, is given by

$$\begin{aligned} l(t)=\frac{l_0 e^{Q(t)-Q(t_0)}}{1-\frac{\eta }{C}l_0\left( 1-e^{Q(t)-Q(t_0)}\right) }, \qquad t\ge t_0, \end{aligned}$$

where $Q(t)-Q(t_0)=\int _{t_0}^tP(\tau )d\tau$. With the hypotesis that $Q(t)\rightarrow +\infty$ when $t\rightarrow \infty$, the carrying capacity of this generalized model is given by $\frac{C}{\eta }$, and thus it is independent from the initial condition $l_0$. In order to obtain a generalized logistic function in which the carrying capacity is dependent on the initial condition, we consider the following equation (cf. in Di Crescenzo et al. (2021))

1 $$\begin{aligned} \frac{d}{dt}l_m(t)=h_\theta (t)l_m(t), \qquad t\ge t_0, \end{aligned}$$

with

2 $$\begin{aligned} h_\theta (t):=\frac{P_\beta (t)e^{-Q_\beta (t)}}{\eta +e^{-Q_\beta (t)}}, \end{aligned}$$

for $\eta >0$, $\theta =(\eta ,\beta ^T)^T$ with $\beta ^T=(\beta _1,\dots , \beta _p)\in {\mathbb {R}}^p$, where

3 $$\begin{aligned} Q_\beta (t)=\sum _{i=1}^p \beta _it^i,\qquad \beta _p>0, \end{aligned}$$

and $P_\beta (t)=\displaystyle \frac{d}{dt}Q_\beta (t)$. Under these assumptions, the solution of the ordinary differential equation (1), with initial condition $l_m(t_0)=l_0$, is the so-called multisigmoidal logistic function given by

4 $$\begin{aligned} l_m(t)=l_0\frac{\eta + e^{-Q_\beta (t_0)}}{\eta + e^{-Q_\beta (t)}}, \qquad t\ge t_0. \end{aligned}$$

**Fig. 1:**
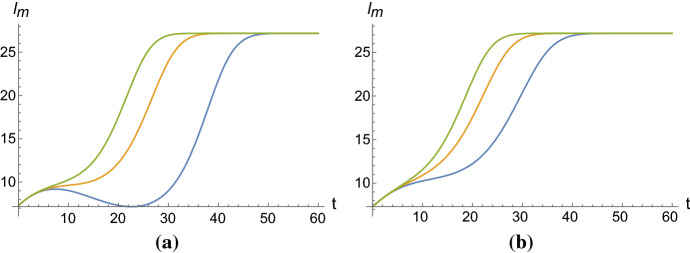
The multisigmoidal logistic function for some choices of the parameters: $t_0=0$, $l_0=\frac{10}{1+\eta }$, $\eta =e^{-1}$, $\beta _1=0.1$, **a** $\beta _2=-0.009$ and, from bottom to top, $\beta _3=0.0002, 0.0003, 0.0004$; **b** $\beta _2=-0.007$ and, from bottom to top, $\beta _3=0.0002, 0.0003, 0.0004$

We point out that the function $l_m$ may exhibit more than one inflection point, and its carrying capacity is

5 $$\begin{aligned} \lim _{t\rightarrow \infty }l_m(t)=l_0\frac{\eta +e^{-Q_\beta (t_0)}}{\eta }=\frac{C}{\eta }, \end{aligned}$$

where $C=C(l_0,\eta ,\beta , t_0)=l_0\left( \eta +e^{-Q_\beta (t_0)}\right)$ and $Q_\beta$ is defined in Eq. (3). It is easy to note that the function (4) is not monotonous in general, since the monotonicity intervals depend on the coefficients $\beta _1,\dots ,\beta _p$ of the polynomial $Q_\beta$, and the carrying capacity is the maximum value attainable by the function $l_m$. See Fig. 1 for some plots of the multisigmoidal logistic function.

The investigation of the inflection points in the case of multisigmoidal growth curves are of great interest. Unfortunately, since the expression of function (4) is quite complex, these points cannot be obtained explicitly, but it is possible to provide an equation in the unknown *t* solved by the inflection points, that is

6 $$\begin{aligned} \frac{d}{dt}P_\beta (t)=P_\beta ^2(t)\left[ \frac{\eta -e^{-Q_\beta (t)}}{\eta +e^{-Q_\beta (t)}}\right] . \end{aligned}$$

**Fig. 2:**
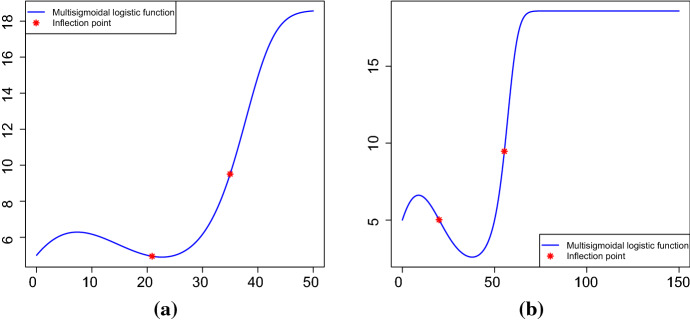
The multisigmoidal logistic function and the corresponding inflection points for $t_0=0$, $l_0=5$, $\eta =e^{-1}$, $\beta _1=0.1$, **a** $\beta _2=-0.009$ and $\beta _3=0.0002$; **b** $\beta _2=-0.007$ and $\beta _3=0.0001$

In Fig. 2, the multisigmoidal logistic function and the corresponding inflection points are shown for some choices of the parameters.

### The corresponding diffusion process

In Di Crescenzo et al. (2021), a special time-dependent lognormal diffusion process $\left\{ X(t);t\in I\right\}$ has been considered, with $I=[t_0,+\infty )$ and infinitesimal moments

7 $$\begin{aligned} A_1(x,t)=h_\theta (t)x,\qquad A_2(x)=\sigma ^2x^2, \end{aligned}$$

where $h_\theta$ is defined in (2), $\theta = (\eta ,\beta ^T )^T$ and $\sigma >0$. The aforementioned process is determined by the following stochastic differential equation, obtained from Eq. (1) by adding a multiplicative noise term,

8 $$\begin{aligned} dX(t)=h_\theta (t)X(t)dt+\sigma X(t)dW(t), \qquad {X(t_0){\mathop {=}\limits ^{d}}X_0}, \end{aligned}$$

where ${\mathop {=}\limits ^{d}}$ means equality in distribution, and where *W*(*t*) denotes a Wiener process independent from the (possibly random) initial state $X_0$, for $t\ge t_0$. We point out that this is not the only way to randomize the growth deterministic equation. Indeed, in the case of random catastrophes, it may be more appropriate to consider as a noise term a Poisson process (see for example Schlomann (2018)). The solution of Eq. (8) is

9 $$\begin{aligned} X(t)=X_0\exp \left[ H_\xi (t_0,t)+\sigma \left( W(t)-W(t_0)\right) \right] ,\qquad t\ge t_0 \end{aligned}$$

with

10 $$\begin{aligned} H_\xi (t_0,t)=\int _{t_0}^{t}h_\theta (\tau )d\tau -\frac{\sigma ^2}{2}(t-t_0) =\log \left[ \frac{\eta +e^{-Q_\beta (t_0)}}{\eta +e^{-Q_\beta (t)}}\right] -\frac{\sigma ^2}{2}(t-t_0). \end{aligned}$$

The existence and uniqueness of solution of the linear stochastic differential equation (8) is ensured by virtue of the continuity of function $h_{\theta }(t)$ (see, for example, Arnold (1974)). Moreover, if either $X_0$ is degenerated at $x_0$, in the sense that ${\mathbb {P}}\left[ X(t_0)=x_0\right] =1$, or $X_0$ follows a lognormal distribution $\varLambda _1\left( \mu _0,\sigma _0^2\right)$, then the finite dimensional distributions of the process are lognormal. Namely, for any $n\in {\mathbb {N}}$ and $t_0\le t_1<\ldots <t_n$, the vector $\left( X(t_1),\dots , X(t_n)\right) ^T$ follows an *n*-dimensional lognormal distribution $\varLambda _n\left( \epsilon , \Sigma \right)$, where the entries of the vector $\epsilon$ are given by

$$\begin{aligned} \epsilon _i=\mu _0+H_\xi (t_0,t_i)=\mu _0+\log \left[ \frac{\eta +e^{-Q_\beta (t_0)}}{\eta +e^{-Q_\beta (t_i)}}\right] -\frac{\sigma ^2}{2}(t_i-t_0),\qquad i=1,\dots ,n, \end{aligned}$$

and the components of the matrix $\Sigma =(\sigma _{i,j})$ are given by

$$\begin{aligned} \sigma _{i,j}=\sigma _0^2+\sigma ^2\left( \min \left( t_i,t_j\right) -t_0\right) ,\qquad i,j=1,\dots ,n. \end{aligned}$$

Further, the conditional distribution of the process follows a lognormal distribution, i.e. for $s<t$

$$\begin{aligned} \left[ X(t)|X(s)=z\right] \sim \varLambda _1\left( \log z+\log \left[ \frac{\eta +e^{-Q_\beta (s)}}{\eta +e^{-Q_\beta (t)}}\right] -\frac{\sigma ^2}{2}(t-s), \sigma ^2(t-s) \right) . \end{aligned}$$

From the above mentioned distributions, some characteristics associated to the process can be obtained (cf. Di Crescenzo et al. (2021)). For example, the mean of *X*(*t*) conditional on $X(t_0)=x_0$ is given by

11 $$\begin{aligned} m(t|t_0)=\mathbb E\left[ X(t)|X(t_0)=x_0\right] =x_0\frac{\eta +e^{-Q_\beta (t_0)}}{\eta +e^{-Q_\beta (t)}}, \qquad t\ge t_0. \end{aligned}$$

Moreover, if $X(t_0){\mathop {=}\limits ^{d}}X_0$ then the mean of *X*(*t*) is

12 $$\begin{aligned} m(t)={\mathbb {E}}\left[ X(t)\right] =\mathbb E\left[ X_0\right] \frac{\eta +e^{-Q_\beta (t_0)}}{\eta +e^{-Q_\beta (t)}},\qquad t\ge t_0, \end{aligned}$$

and the $\alpha$-percentiles for $t\ge t_0$ are

13 $$\begin{aligned} C_\alpha [X(t)]=\frac{\eta +e^{-Q_\beta (t_0)}}{\eta +e^{-Q_\beta (t)}}\exp \left( \mu _0-\frac{\sigma ^2}{2}(t-t_0)+z_\alpha \sqrt{\sigma _0^2+\sigma ^2(t-t_0)}\right) , \end{aligned}$$

for $0<\alpha <1$, where $z_\alpha$ is the $\alpha$-percentile of the standard normal random variable. Note that the conditional mean (11) and the mean (12) are multisigmoidal logistic functions of *t*, in the sense that they solve the multisigmoidal logistic equation (1).

## Maximum likelihood estimations

The stochastic model introduced in Sect. 2.1 can be employed in several applications, especially for describing real populations that exhibit a growth pattern with more than one inflection point. Clearly, in order to apply this model to real data, the unknown parameters need to be estimated. In Sect. 2.1 we obtained the distribution of the diffusion process *X*(*t*) defined in (9). Now we propose to estimate the parameters by means of the classical maximum likelihood method. The adoption of this strategy is particularly suggested by the availability in closed form of the transition distribution of the process *X*(*t*). Hence, we follow the same lines introduced in Román-Román et al. (2018) for general lognormal diffusion processes. We consider a discrete sampling of *X*(*t*) based on *d* independent sample paths, with $n_i$ different observation instants for the *i*-th sample path, i.e. $t_{ij}$, $j=1,\dots , n_i$, for $i=1,\dots ,d$. For simplicity, assume that the first observation time is identical for any trajectory, i.e. $t_{i1}=t_0$, $i=1,\dots ,d$. Moreover, let the vector ${\mathbb {X}}_i=\left( X(t_{i1}),\dots , X(t_{in_i})\right) ^T$ contain the variables of the *i*-th sample path, for $i=1,\dots , d$, and let ${\mathbb {X}}=\left( {\mathbb {X}}_1^T|\dots | \mathbb X_d^T\right) ^T$. By supposing that $X(t_0)$ follows a one-dimensional lognormal distribution $\varLambda _1\left( \mu _1,\sigma _1^2\right)$ and by considering the transitions of the process *X*(*t*), the probability density function of ${\mathbb {X}}$ has the following expression

14 $$\begin{aligned} f_{{\mathbb {X}}}(x)=\prod _{i=1}^{d}\exp \left( -\frac{\left( \log x_{i1}-\mu _1\right) ^2}{x_{i1}\sigma _1 \sqrt{2\pi }}\right) \cdot \prod _{j=1}^{n_i-1}\frac{\exp \left( -\frac{\left[ \log \left( \frac{x_{i,j+1}}{x_{ij}}\right) -m_{\xi }^{i,j+1,j}\right] ^2}{2\sigma ^2\varDelta _i^{j+1,j}}\right) }{x_{ij}\sigma \sqrt{2\pi \varDelta _{i}^{j+1,j}}},\nonumber \\ \end{aligned}$$

where $x^T=\left( x_{1,1},\dots ,x_{1,n_1}|\dots |x_{d,1},\dots ,x_{d,n_d}\right) \in {\mathbb {R}}_+^{n+d}$ is a vector of dimension $n+d$, with

15 $$\begin{aligned} n=\sum _{i=1}^d (n_i-1). \end{aligned}$$

Recalling (10), the parameters in (14) are given by

16 $$\begin{aligned} m_{\xi }^{i,j+1,j}:=H_\xi \left( t_{ij},t_{i,j+1}\right) =\log \left[ \frac{\eta +e^{-Q_{\beta }(t_{ij})}}{\eta +e^{-Q_{\beta }(t_{i,j+1})}}\right] -\frac{\sigma ^2}{2}\varDelta _{i}^{j+1,j}, \end{aligned}$$

and

$$\begin{aligned} \varDelta _i^{j+1,j}:=t_{i,j+1}-t_{ij},\qquad j=1,\dots ,n_i-1,\quad i=1,\dots ,d, \end{aligned}$$

with

$$\begin{aligned} \xi =\left( \theta ^T,\sigma ^2\right) ^T=\left( \eta ,\beta ^T,\sigma ^2\right) ^T. \end{aligned}$$

In order to obtain a more manageable expression of the density (14), the following change of variables may be considered:

$$\begin{aligned} \begin{aligned}&V_{0i}=X_{i1}, \qquad i=1,\dots ,d\\&V_{ij}=\left( \varDelta _i^{j+1,j}\right) ^{-1/2}\log \frac{X_{i,j+1}}{X_{ij}},\qquad j=1,\dots ,n_i-1,\;\; i=1,\dots , d. \end{aligned} \end{aligned}$$

Hence, the probability density function of the vector $\mathbb V=\left[ {\mathbb {V}}_0^T|{\mathbb {V}}_1^T|\dots |\mathbb V_d^T\right] ^T=\left[ {\mathbb {V}}_0^T|{\mathbb {V}}_{(1)}^T\right] ^T$, with ${\mathbb {V}}_{(1)}^T=\left( {\mathbb {V}}_1^T|\dots |{\mathbb {V}}_d^T\right)$, and ${\mathbb {V}}_i^T=(V_{i1}, V_{i2}, \ldots , V_{i n_i})$, is

$$\begin{aligned}&f_{{\mathbb {V}}}(v)=\frac{\exp \left[ -\frac{1}{2\sigma _1^2} \left( lv_0-\mu _1{\mathbb {I}}_d\right) ^T\left( lv_0-\mu _1\mathbb I_d\right) \right] }{\prod _{i=1}^d v_{0i}\left( 2\pi \sigma _1^2\right) ^{d/2}}\\&\quad \cdot \frac{\exp \left[ -\frac{1}{2\sigma ^2}\left( v_{(1)}-\gamma ^\xi \right) ^T\left( v_{(1)}-\gamma ^\xi \right) \right] }{\left( 2\pi \sigma ^2\right) ^{n/2}} \end{aligned}$$

for $v=(v_{01},\dots , v_{0d}, v_{11},\dots , v_{{1},{n_1-1}},\ldots , v_{d1},\dots , v_{d,{n_d-1}})^T\in {\mathbb {R}}^{n+d}$, with *n* defined in (15), where $lv_0=\left( \log v_{01},\dots ,\log v_{0d}\right) ^T$, and $\mu _1\in {\mathbb {R}}$, $\sigma _1^2\in \mathbb R_+$, ${\mathbb {I}}_d=(1,\dots ,1)^T_{d\times 1}$, with $\gamma ^\xi =(\gamma ^\xi _{11},\ldots , \gamma ^\xi _{1,n_1-1},\ldots , \gamma ^\xi _{d1},\ldots , \gamma ^\xi _{d,n_d-1})^T \in \mathbb R^{n\times 1}$ and $\gamma ^\xi _{ij}=\left( \varDelta _i^{j+1,j}\right) ^{-1/2}m_\xi ^{i,j+1,j}$, for $j=1,\dots ,n_i-1$ and $i=1,\dots ,d$.

By setting $\alpha =\left( \mu _1,\sigma _1^2\right) ^T$ and supposing that $\alpha$ and $\xi$ are functionally independent, the log-likelihood function is given by

17 $$\begin{aligned} L_{{\mathbb {V}}}\left( \alpha ,\xi \right)= & {} {{\tilde{L}}}_{\mathbb V}(\xi )-\frac{(n+d)}{2}\log 2\pi -\frac{d}{2}\log \sigma _1^2-\sum _{i=1}^d \log v_{0i}\nonumber \\&-\frac{\sum _{i=1}^d\left( \log v_{0i}-\mu _1\right) ^2}{2\sigma _1^2}, \end{aligned}$$

with

$$\begin{aligned} {{\tilde{L}}}_{{\mathbb {V}}}(\xi )=-\frac{n}{2}\log \sigma ^2-\frac{Z_1+\varPhi _\xi -2\varGamma _\xi }{2\sigma ^2} \end{aligned}$$

and

$$\begin{aligned} Z_1=\sum _{i=1}^d\sum _{j=1}^{n_i-1}v_{ij}^2,\qquad \varPhi _\xi =\sum _{i=1}^d\sum _{j=1}^{n_i-1}\frac{\left( m_\xi ^{i,j+1,j}\right) ^2}{\varDelta _i^{j+1,j}},\qquad \varGamma _\xi =\sum _{i=1}^d\sum _{j=1}^{n_i-1}\frac{v_{ij}m_\xi ^{i,j+1,j}}{\left( \varDelta _i^{j+1,j}\right) ^{1/2}}. \end{aligned}$$

The maximum likelihood estimations (MLEs) of $\alpha =\left( \mu _1,\sigma _1^2\right) ^T$ can be computed easily. Indeed, by differentiating $L_{{\mathbb {V}}}$, from (17) we obtain

18 $$\begin{aligned} {\hat{\mu }}_{1}=\frac{1}{d}\sum _{i=1}^d \log v_{0i},\qquad {\hat{\sigma }}_{1}^{2}=\frac{1}{d}\sum _{i=1}^d \left( \log v_{0i}-{\hat{\mu }}_{1}\right) ^{2}. \end{aligned}$$

Further on, in order to find the maximum likelihood estimates of $\xi$, two different approaches are available:

(i) solving the nonlinear system $\frac{\partial }{\partial \xi }{{\tilde{L}}}_{{\mathbb {V}}}=0,$
(ii) maximizing the objective function ${{\tilde{L}}}_{{\mathbb {V}}}$.

Hereafter, in the Sects. 3.1 and 3.2 we provide a description of the two strategies, whereas in Sect. 5 we present an application to a simulation study that involves the given strategies.

The availability of the probability density function of ${\mathbb {X}}$ in (14) allows to obtain explicitly the log-likelihood function given in (17). Consequently, following the maximum likelihood estimation procedure, in Sect. 3.1 we obtain the associated system of equations, the final form being reported in Eq. (23) below. However, since such system does not have an explicit solution, its resolution must be obtained by adopting numerical methods.

### Solving the nonlinear system

Recalling that $\theta ^T=(\theta _0,\theta _1,\dots ,\theta _p)=(\eta ,\beta _1,\dots ,\beta _p)$, the partial derivatives of ${{\tilde{L}}}_{{\mathbb {V}}}$ are given by

$$\begin{aligned}&\frac{\partial }{\partial \sigma ^2}{{\tilde{L}}}_{{\mathbb {V}}} =-\frac{n}{2\sigma ^2}+\frac{Z_1+\varPhi _\xi -2\varGamma _\xi }{2\sigma ^4}+\frac{Y_\xi }{2\sigma ^2}-\frac{Z_2}{2\sigma ^2}, \\&\quad \frac{\partial }{\partial \theta }{{\tilde{L}}}_{\mathbb V}=-\frac{1}{2\sigma ^2}\left[ \frac{\partial }{\partial \theta }\varPhi _\xi -2\frac{\partial }{\partial \theta }\varGamma _\xi \right] , \end{aligned}$$

where

$$\begin{aligned}&Y_\xi :=\sum _{i=1}^d\sum _{j=1}^{n_i-1}m_\xi ^{i,j+1,j},\qquad Z_2:=\sum _{i=1}^d\sum _{j=1}^{n_i-1}v_{ij}\left( \varDelta _i^{j+1,j}\right) ^{1/2}, \\&\quad \frac{\partial }{\partial \theta } \varPhi _\xi =\sum _{i=1}^{d}\sum _{j=1}^{n_i-1}\frac{2m_\xi ^{i,j+1,j}\frac{\partial }{\partial \theta }m_\xi ^{i,j+1,j}}{\varDelta _i^{j+1,j}}, \\&\quad \frac{\partial }{\partial \theta }\varGamma _\xi =\sum _{i=1}^d\sum _{j=1}^{n_i-1} \frac{v_{ij}}{\left( \varDelta _i^{j+1,j}\right) ^{1/2}}\cdot \frac{\partial }{\partial \theta }m_\xi ^{i,j+1,j}, \end{aligned}$$

with

$$\begin{aligned} \frac{\partial }{\partial \theta }m_\xi ^{i,j+1,j}=\left( \frac{\partial }{\partial \theta _0},\dots ,\frac{\partial }{\partial \theta _p}\right) m_{\xi }^{i,j+1,j}. \end{aligned}$$

Hence, the MLEs are the solutions of the following system of $p+2$ nonlinear equations

19 $$\begin{aligned} {\left\{ \begin{array}{ll} &{}-n\sigma ^2+Z_1+\varPhi _\xi -2\varGamma _\xi +Y_\xi \sigma ^2-Z_2\sigma ^2=0, \\ &{}\displaystyle {\sum _{i=1}^d\sum _{j=1}^{n_i-1}\frac{m_\xi ^{i,j+1,j}}{\varDelta _i^{j+1,j}}\cdot \frac{\partial }{\partial \theta }m_\xi ^{i,j+1,j}-\sum _{i=1}^d\sum _{j=1}^{n_i-1}\frac{v_{ij}}{\left( \varDelta _i^{j+1,j} \right) ^{1/2}}\cdot \frac{\partial }{\partial \theta }m_\xi ^{i,j+1,j}=0.} \end{array}\right. }\nonumber \\ \end{aligned}$$

By defining the following quantities

20 $$\begin{aligned} \begin{aligned}&_{l}\varDelta _{\theta }^{i,m}:=\frac{1}{\eta +e^{-Q_\beta (t_{im})}}\left( -t_{im}^l\right) ,\\&{\bar{\delta }}_{l0}:=1-\delta _{l0}= {\left\{ \begin{array}{ll} 0,\quad l=0\\ 1,\quad l\ne 0 \end{array}\right. },\\&_lD_\theta ^{i,m,n}:={_l\varDelta _\theta ^{i,m}\left( e^{-Q_\beta (t_{im})}\right) ^{{\bar{\delta }}_{l0}}}-{_l\varDelta _\theta ^{i,n}\left( e^{-Q_\beta (t_{in})}\right) ^{{\bar{\delta }}_{l0}}},\quad m>n, \end{aligned} \end{aligned}$$

with $l=0,1,\dots ,p$, the last $p+1$ equations of the system (19) can be written as follows

$$\begin{aligned}&-\sum _{i=1}^d\sum _{j=1}^{n_i-1}\frac{m_\xi ^{i,j+1,j}}{\varDelta _i^{j+1,j}}{_lD_\theta ^{i,j+1,j}}+\sum _{i=1}^d\sum _{j=1}^{n_i-1}\frac{v_{ij}}{\left( \varDelta _i^{j+1,j}\right) ^{1/2}}{_lD_\theta ^{i,j+1,j}}=0,\\&\qquad l=0,1,\dots ,p. \end{aligned}$$

Substituting the expression (16) of $m_\xi$ in the previous equations, one has

$$\begin{aligned} Y_l^\theta +\frac{\sigma ^2}{2}W_l^\theta +X_l^\theta =0,\qquad l=0,1,\dots ,p, \end{aligned}$$

where, for any $l=0,1,\dots ,p$, one has

$$\begin{aligned} \begin{aligned}&W_l^\theta :=\sum _{i=1}^d\sum _{j=1}^{n_i-1} {_lD_\theta ^{i,j+1,j}}=\sum _{i=1}^d {_lD_\theta ^{i,n_i,1}}, \\&Y_l^\theta :=\sum _{i=1}^d\sum _{j=1}^{n_i-1} \frac{1}{\varDelta _i^{j+1,j}}\log \left[ \frac{\eta +e^{-Q_\beta (t_{i,j+1})}}{\eta +e^{-Q_\beta (t_{ij})}}\right] {_lD_\theta ^{i,j+1,j}}, \\&X_l^\theta :=\sum _{i=1}^d\sum _{j=1}^{n_i-1}\frac{v_{ij}}{\left( \varDelta _{i}^{j+1,j}\right) ^{1/2}}{_lD_\theta ^{i,j+1,j}}. \end{aligned} \end{aligned}$$

Hence, until now, the expression of the system solved by the MLEs is

21 $$\begin{aligned} {\left\{ \begin{array}{ll} -n\sigma ^2+Z_1+\varPhi _\xi -2\varGamma _\xi +Y_\xi \sigma ^2-Z_2\sigma ^2=0\\ Y_l^\theta +\frac{\sigma ^2}{2}W_l^\theta +X_l^\theta =0,\qquad \qquad l=0,1,\dots ,p. \end{array}\right. } \end{aligned}$$

The first equation of system (21) can be further simplified. Indeed, by setting

22 $$\begin{aligned} \begin{aligned}&\lambda _\theta ^{i,m,n}:=\log \frac{\eta +e^{-Q_\beta (t_{in})}}{\eta +e^{-Q_\beta (t_{im})}},\quad m>n\qquad Z_3:=\sum _{i=1}^d \varDelta _i^{n_{i,1}},\\&A_\theta :=\sum _{i=1}^d\sum _{j=1}^{n_i-1}\frac{\left( \lambda _\theta ^{i,j+1,j}\right) ^2}{\varDelta _i^{j+1,j}},\quad B_\theta :=\sum _{i=1}^d\sum _{j=1}^{n_i-1}\frac{v_{ij}\lambda _\theta ^{i,j+1,j}}{\left( \varDelta _i^{j+1,j}\right) ^{1/2}},\\&C_\theta :=\sum _{i=1}^d\sum _{j=1}^{n_i-1}\lambda _\theta ^{i,j+1,j}, \end{aligned} \end{aligned}$$

one has

$$\begin{aligned} \varPhi _\xi =A_\theta +\frac{\sigma ^4}{4}Z_3-\sigma ^2C_\theta , \qquad \varGamma _\xi =B_\theta -\frac{\sigma ^2}{2}Z_2, \qquad Y_\xi =C_\theta -\frac{\sigma ^2}{2}Z_3. \end{aligned}$$

Consequently, the system (21) finally becomes

23 $$\begin{aligned} {\left\{ \begin{array}{ll} \sigma ^2\left( n+\frac{\sigma ^2}{4}Z_3\right) -Z_1-A_\theta +2B_\theta =0\\ Y_l^\theta +\frac{\sigma ^2}{2}W_l^\theta +X_l^\theta =0,\qquad l=0,1,\dots ,p. \end{array}\right. } \end{aligned}$$

Note that (23) is a system of $p+2$ equations in the unknowns contained in $\xi =(\eta ,\beta _1,\dots ,\beta _p,\sigma ^2)^T$.

#### Remark 1

For the first equation of the system (23) in the unknown $\sigma ^2$, since $Z_3>0$, $n>0$ and

$$\begin{aligned} Z_1+A_\theta -2B_\theta =\sum _{i=1}^{d}\sum _{j=1}^{n_i}\left( v_{ij}-\frac{\lambda _\theta ^{i,j+1,j}}{\left( \varDelta _{i}^{j+1,j}\right) ^{1/2}}\right) ^2\ge 0, \end{aligned}$$

the only acceptable solution is

$$\begin{aligned} \sigma ^2={2}\,\frac{-n+\sqrt{n^2+Z_3(Z_1+A_\theta -2B_\theta )}}{Z_3}. \end{aligned}$$

Clearly, since in general system (23) cannot be solved analytically, then a numerical approach is needed. Specifically, we adopt the well-known Newton-Raphson method to solve (23) (for instance, see Dennis and Schnabel (1996)). For such an iterative method, an initial approximation for the solution of the system is needed. It can be obtained by a procedure similar to that used by Román-Román et al. (2019). For the initial solution of the vector $\theta = \left( \eta , \beta _1,\dots ,\beta _p\right) ^T$, by considering the multisigmoidal logistic function, i.e.

$$\begin{aligned} l_m(t)=\frac{C}{\eta +e^{-Q_\beta (t)}},\qquad t\ge t_0, \end{aligned}$$

it can be supposed, without loss of generality, that $t_0=0$ (see Remark 2.1 of Di Crescenzo et al. (2021)), so that

$$\begin{aligned} Q_\beta (t)+\log \eta =-\log \left( \frac{C/\eta }{l_m(t)}-1\right) . \end{aligned}$$

Then, considering the sampling ${\mathbb {X}}=\left( {\mathbb {X}}_1^T| \dots | {\mathbb {X}}_d^T \right) ^T$ defined in Sect. 3, consisting of *d* independent sample paths of the process *X*(*t*), for simplicity we suppose that any sample path of the process has the same number of observations, i.e. $n_i=N$ for any $i=1,\dots , d$. However, the following remarks hold even in more general cases. Moreover, let $m_j$ be the values of the mean of the sample paths at the time $t_j$, for $j=1,\dots , N$, that is

24 $$\begin{aligned} m_j=\frac{1}{d}\sum _{i=1}^{d} x_{ij}, \qquad j=1,2,\dots ,N, \end{aligned}$$

where $x_{ij}$ is the value of the *i*-th sample path at the time $t_j$.

In general, the carrying capacity $C/\eta$ is unknown. We suppose that the observations are available over a large time interval, such that the evolution of the population is terminated over such an interval. Hence, the carrying capacity $C/\eta$ can be approximated with the last value of the sample mean $m_N$. This approximation can be adopted also in the other cases, since it is used just to construct an initial solution for the parameters of the Newton-Raphson method for the estimate of $\theta$. Thus, we can consider a polynomial regression for the pairs

$$\begin{aligned} \left( t_j, -\log \left( \frac{m_N}{m_j}-1\right) \right) , \qquad j=1,2,\dots , N-1. \end{aligned}$$

The coefficients $({{\hat{\beta }}}_1,\dots ,{{\hat{\beta }}}_p,\log {{\hat{\eta }}})$ of the approximating polynomial will be the initial values for the parameters $(\beta ^T,\log \eta )$. Thus, the initial solution for $\eta$ is given by ${{\hat{\eta }}}$.

Finally, in order to construct the initial solution of $\sigma ^2$, let us now recall that for a lognormal distribution $Y\sim \varLambda _1(\alpha ,\delta )$, one has $\log Y\sim \mathcal N(\alpha ,\delta )$, so that the quantity $2\log \frac{m}{m^g}$ gives an approximation for $\delta$, where *m* and $m^g$ are respectively the arithmetic sample mean and the geometric sample mean of a random sample $(y_1,\ldots , y_n)$ from *Y*. Hence, one has

$$\begin{aligned} \alpha \approx \frac{1}{n}\sum _{i=1}^n \log y_i, \qquad e^\alpha \approx e^{\frac{1}{n}\sum _{i=1}^n \log y_i}=\left( \prod _{i=1}^n y_i\right) ^{1/n}=m_g. \end{aligned}$$

Since ${\mathbb {E}}[Y]=e^{\alpha +\delta /2}$ is estimated by the sample mean *m*, we have

$$\begin{aligned} m\approx e^{\alpha +\delta /2}\approx m_g \cdot e^{\delta /2}, \qquad \delta \approx 2\log \frac{m}{m_g}. \end{aligned}$$

As a consequence, in our setting an estimate for $\sigma _0^2+\sigma ^2t_j$ is given by

$$\begin{aligned} \sigma _j^2=2\log \frac{m_j}{m_j^g}, \qquad j=1,\dots ,N, \end{aligned}$$

where $m_j$ and $m_j^g$ denote respectively the arithmetic and the geometric sample mean of the observations performed at the time $t_j$. Hence, an initial approximation for $\sigma ^2$ can be obtained by performing a simple linear regression of $\sigma _j^2-\sigma _0^2$ against $t_j$.

In conclusion, in order to obtain the maximum likelihood estimates of the parameters contained in $\xi =(\eta ,\beta _1,\dots ,\beta _p,\sigma ^2)^T$, the steps of the proposed strategy to solve the system (23) are:

(i) finding an initial solution for the parameters $\eta$ and $\beta$ with a polynomial regression of $-\log \left( \frac{m_N}{m_j}-1\right)$ against $t_j$, for any $j=1,\dots , N-1$;
(ii) finding an initial solution for $\sigma ^2$ with a simple linear regression of $\sigma _j^2-\sigma _0^2$ against $t_j$, with $\sigma ^2_j=2\log \frac{m_j}{m_j^g}$, for any $j=1,\dots ,N$ and where $\sigma _0^2$ can be obtained by means of the second of Eqs. (18);
(iii) using the Newton-Raphson method to solve the system (23), with the initial solutions determined at steps (i) and (ii).

The adoption of the above strategy requires to start from good initial solutions for the unknown parameters. Unfortunately, even in this case it is not always possible to guarantee the convergence of this method. For this reason, recently various procedures have been proposed aimed at addressing the maximization of the likelihood function, by viewing this as a direct optimization problem. Indeed, there is a wide range of stochastic metaheuristic methods, which can be classified into two large families: those based on trajectories and those based on swarms. Hereafter, in Sect. 3.2 we employ one of the most widely used, the Simulated Annealing. This method requires necessarily to bound the parametric space, and this matter is the object of Sect. 3.2.2.

### Maximizing the log-likelihood function

Let us now illustrate a strategy based on Simulated Annealing (S.A.) and finalized to obtain the MLEs for the parameters of the process (9). We first provide a brief description of this method in Sect. 3.2.1. Then, in Sect. 3.2.2 we describe a suitable criterion to restrict the parametric space, this being essential to apply the S.A. method in the remainder of the paper.

#### Brief notes on Simulated Annealing

The aim of this section is to determine the MLEs by using the S.A. algorithm. The aforementioned method, introduced in Kirkpatrick et al. (1983), is a meta-heuristic optimization algorithm used for problems like finding $\displaystyle \arg \min _{\theta \in \Theta } f(\theta )$. It is considered more suitable with respect to other numerical algorithms since it needs less restrictive conditions regarding the regularity of the domain $\Theta$ and the analytical properties of the objective function *f*. The algorithm works such that in every step a random point is chosen in the solution space. If the new solution is better than the previous one, then the latter is replaced. Otherwise, if the new solution is worse than the previous, then the latter may be replaced with a probability rate $\rho =\min \{\exp (-\varDelta f/T),1\}$ which depends on the increase of the objective function $\varDelta f=f(\xi )-f(\theta _0)$ and on a suitable scale factor *T*, that is named ‘temperature’ in agreement with the metallurgical process of annealing that inspired this algorithm. We recall that the S.A. is successful because it avoids local minima. In recent years it has been widely used in the context of estimation in diffusion processes (see, for example Luz Sant’Ana et al. (2018) and Román-Román and Torres-Ruiz (2015)).

In this context, the algorithm works in the following way. It begins with an initial choice $\theta _0$ for the parameters of interest, then $\xi$ is generated from an uniform distribution in a neighborhood $\nu (\theta _0)$ of $\theta _0$. Then, a new value $\theta _1$ of $\theta$ is obtained in such a way

$$\begin{aligned} \theta _1={\left\{ \begin{array}{ll} \xi ,&{}\qquad \text { with probability } \rho \\ \theta _0,&{}\qquad \text{ with } \text{ probability } 1-\rho . \end{array}\right. } \end{aligned}$$

Consequently, if $f(\xi )\le f(\theta _0)$, then $\rho =1$ and therefore $\theta _0$ is replaced by $\xi$. Otherwise, if $f(\xi )>f(\theta _0)$, then $\xi$ may be accepted anyway with probability $\rho \in (0,1)$. The temperature *T* is defined in such a way that at the beginning the probability of accepting $\xi$ is high, and during the execution of the algorithm the function *T* decreases. The initial temperature $T_0$ must be sufficiently large so that the algorithm accept the solutions which let the objective function increases with a large probability $p_0$. In literature, the choices of the initial parameters are usually $p_0=0.9$ and $T_0=-\varDelta f^+/\log p_0$, where $\varDelta f^+$ denotes the average increase of the objective function in an application test where all the solutions which cause an increase are accepted. The cooling process which defines the temperature *T* is usually chosen of geometric type, i.e. $T_i=\gamma T_{i-1}$ for $i=1,2,\dots$. Usually the constant $\gamma$ is chosen among 0.8 and 0.99 in order to have a slow cooling procedure. In our case, we set $\gamma =0.95$. In any iteration of the algorithm, a chain of *L* new solutions is obtained, for $L=50$. As required, the algorithm stops when at least one of the following rules is satisfied: (i) the last *L* obtained values are equal, (ii) the maximum number of iterations (1000, in our case) is attained, (iii) the final temperature $T_F=10^{-7}$ is reached.

#### Bounding the parametric space

S.A. needs a restriction of the solution space $\Theta$, namely the set which contains the parameters $\xi =(\eta ,\beta ^T,\sigma ^2)^T$. Until now, this space is continuous and unbounded, since

$$\begin{aligned} \Theta =\left\{ {(\eta ,\beta ^T,\sigma ^2)}^{T}: \eta>0, \beta _1,\dots ,\beta _{p-1}\in {\mathbb {R}}, \beta _p>0, \sigma ^2>0\right\} . \end{aligned}$$

We consider $0<\sigma <0.1$ so that the simulated sample paths are less variable around the sample mean, and thus the multisigmoidal logistic profile is advisable. For the parameters $\beta =\left( \beta _1,\dots ,\beta _p\right) ^T$, we find the confidence intervals by using the data of the polynomial regression performed previously to find the initial solutions. More in detail, it is known that the carrying capacity of the multisigmoidal logistic model with $t_0=0$ is $l_0 \left( 1+\frac{1}{\eta }\right)$ (see Eq. (5)). The carrying capacity can be approximated with the last value of the sample mean, whereas the initial value $l_0$ with the first value of the sample mean (24), so that one has

25 $$\begin{aligned} m_N\approx m_1\left( 1+\frac{1}{\eta }\right) . \end{aligned}$$

From Eq. (25), it easily follows $\eta \approx \left( \frac{m_N}{m_1}-1\right) ^{-1}$ and thus an approximation of $\eta$ is

$$\begin{aligned} {{\hat{\eta }}}=\left( \frac{m_N}{m_1}-1\right) ^{-1}. \end{aligned}$$

Considering Eqs. (4) and (5), for $t_0=0$ one has

$$\begin{aligned} l_m(t)= \frac{C}{\eta +e^{-Q_\beta (t)}}, \end{aligned}$$

so that

$$\begin{aligned} Q_\beta (t)=-\log \left( \frac{C}{l_m(t)}-\eta \right) . \end{aligned}$$

Hence, by replacing $\eta$ with its estimate ${{\hat{\eta }}}$, we can use the resulting confidence intervals of the parameters of the polynomial regression as intervals of variation for the parameter $\beta$ of the diffusion process. We adopt a confidence level equal to 0.999, to attain a high probability that the true parameter $\beta$ belong to the computed intervals.

In order to approximate the range of variation of $\eta$, from Eq. (25) we have that the last value of the *i*-th sample path satisfies

$$\begin{aligned} x_{i,n_i}\approx x_{i,1}+\frac{x_{i,1}}{\eta }, \qquad i=1,2,\dots ,d, \end{aligned}$$

where $x_{i,j}$ with $i=1,2,\dots ,d$ and $j=1,2,\dots ,n_i$ are the sample data. Hence, for the range of variation of $\eta$ one has $\eta \in (a,b)$, where

26 $$\begin{aligned} a:=\min _{1\le i\le d}\left( \frac{x_{i,n_i}}{x_{i,1}}-1\right) ^{-1}, \qquad b:=\max _{1\le i\le d}\left( \frac{x_{i,n_i}}{x_{i,1}}-1\right) ^{-1}. \end{aligned}$$

In conclusion, the following bounded intervals are employed:

- for $\beta _1,\dots ,\beta _p$ we consider the confidence intervals of the coefficients of the polynomial regression of $-\log \left[ \left( \frac{m_N}{m_j}-1\right) {{\hat{\eta }}}\right]$ against $t_j$, for $j=1,\dots ,N$, where ${{\hat{\eta }}}=\displaystyle \left( \frac{m_N}{m_1}-1\right) ^{-1}$,
- for $\eta$ we consider the interval $I_\eta =\left( a,b\right)$, with *a* and *b* defined in (26),
- for $\sigma ^2$ we consider the interval $I_{\sigma ^2}=\left( 0,0.01\right)$.

### Asymptotic distribution of the MLEs

On the ground of the results given in Sect. 5 of Román-Román et al. (2018), in this section we aim to determine the asymptotic distribution of the MLEs (i) of the parameters $\mu _1,\sigma _1^2$ of the initial distribution, and (ii) of the parameters $\xi =(\eta ,\beta ^T,\sigma ^2)^T$ of the process.

(i) The exact distribution of ${{\widehat{\mu }}}_1$ is normal ${\mathcal {N}}\left( \mu _1,\frac{\sigma _1^2}{d}\right)$, whereas the exact distribution of $d\,\frac{{{\widehat{\sigma }}}_1^2}{\sigma _1^2}$ is chi-square $\chi _{d-1}^2$, cf. Román-Román et al. (2018).
(ii) The asymptotic distribution of ${{\widehat{\xi }}}$ is a $(p+2)$-dimensional normal distribution with mean $\xi$ and covariance matrix $I(\xi )^{-1}$, i.e. ${\mathcal {N}}_{p+2}\left( \xi , I(\xi )^{-1}\right)$, where $I(\xi )$ denotes Fisher’s information matrix of $\xi$. For the diffusion process *X*(*t*) with a multisigmoidal logistic mean,

$I(\xi )\in {\mathbb {R}}^{(p+2)\times (p+2)}$ can be expressed as

27 $$\begin{aligned} I(\xi )=\frac{1}{\sigma ^2}\begin{pmatrix} \Xi _\xi &{} -\frac{1}{2}\left( \frac{\partial }{\partial \theta }\gamma _{\xi } \right) \\ -\frac{1}{2}\left( \frac{\partial }{\partial \theta }\gamma _{\xi }\right) ^T&{} \frac{n}{2\sigma ^2}-\frac{Z_3}{4}, \end{pmatrix}, \end{aligned}$$

where $\Xi _\xi \in {\mathbb {R}}^{(p+1)\times (p+1)}$ is given by

$$\begin{aligned} \Xi _\xi =\sum _{i=1}^{d}\sum _{j=1}^{n_i-1}(\varDelta _i^{j+1,j})^{-1}\left( \frac{\partial }{\partial \theta }m_\xi ^{i,j+1,j}\right) \left( \frac{\partial }{\partial \theta }m_\xi ^{i,j+1,j}\right) ^T, \end{aligned}$$

with

$$\begin{aligned} \left( \frac{\partial }{\partial \theta }m_\xi ^{i,j+1,j}\right) ^T=\left( -{_0D_\theta ^{i,j+1,j}},\dots ,-{_pD_\theta ^{i,j+1,j}}\right) , \end{aligned}$$

and ${_lD_\theta }^{i,j+1,j}$ is defined in the third of Eqs. (20). Moreover, $\frac{\partial }{\partial \theta }\gamma _{\xi }\in {\mathbb {R}}^{(p+1)\times 1}$ is defined as

$$\begin{aligned} \frac{\partial }{\partial \theta }\gamma _{\xi }=\sum _{i=1}^d\sum _{j=1}^{n_i-1}\frac{\partial }{\partial \theta }m_\xi ^{i,j+1,j}. \end{aligned}$$

Finally, $Z_3$ is given in the second of Eqs. (22). We point out that the matrix (27) will be used in Sect. 5 to determine the asymptotic variances for the estimates of the parameters and an approximation of the confidence intervals. Indeed, by applying the delta method (cf. Oehlert (1992)), any *q*-parametric function $g({{\hat{\xi }}})$ with $q\le p+2$ asymptotically has a *q*-dimensional normal distribution, i.e. (cf. Román-Román et al. (2018))

$$\begin{aligned} {\mathcal {N}}_{q}\left( g(\xi ), \nabla g(\xi )^T I(\xi )^{-1}\nabla g(\xi )\right) , \end{aligned}$$

where $\nabla g(\xi )$ is the vector of partial derivatives of $g(\xi )$ with respect to $\xi$.

In the following section we address a relevant problem for the applications, namely the FPT problem of the diffusion process *X*(*t*) through a continuous boundary. Subsequently, in Sect. 5 we adopt a simulation-based approach as the basis of both computational methods described so far, namely the Newton-Raphson method and the S.A. method. The estimates of the parameters obtained through these methods are then used to perform inference on the FPT density.

## First-passage-time problem

The FPT problem of a stochastic process *X*(*t*) through a boundary *S*(*t*) is a problem of great interest in many fields of application, such as medicine, biology or mathematical finance, since the threshold *S*(*t*) may represent a critical value of the modeled population size. Considering a stochastic process $\left\{ X(t);t_0\le t\le T\right\}$, the FPT of the process *X*(*t*) through the continuous boundary *S*(*t*), given $X(t_0)=x_0$, is defined as the following random variable

$$\begin{aligned} T={\left\{ \begin{array}{ll} \displaystyle \inf _{t\ge t_0}\left\{ X(t)>S(t)|X(t_0)=x_0\right\} , \quad x_0<S(t_0)\\ \displaystyle \inf _{t\ge t_0}\left\{ X(t)<S(t)|X(t_0)=x_0\right\} , \quad x_0>S(t_0). \end{array}\right. } \end{aligned}$$

Finding the expression of the distribution of the variable *T* is hard in general. However, in literature there are several studies for particular types of processes, for example diffusion processes. It has been shown that if *S*(*t*) is a continuous and differentiable function, then the density of *T*, denoted by $g\left( S(t),t|x_0,t_0\right)$, solves a II-kind Volterra equation (cf. Eq. (2.4) of Buonocore et al. (1987)). The aforementioned Volterra equation has an explicit solution only for certain special boundaries (see for example Sects. 2.3 and 4.3 of Giorno and Nobile (2019) in which the FPT density through special boudaries has been obtained for the restricted Gompertz-type diffusion processes). In certain instances, it is appropriate to adopt numerical procedures in order to approximate its solution. To this aim Buonocore et al. (1987) proposed a simple but efficient algorithm, based on the composite trapezoidal formula. More in detail, Theorem 4 of Buonocore et al. (1987) proves the convergence of the approximated FPT density to the theoretical one. However, the application of the proposed numerical procedure requires (i) the choice of a suitable step *h* of integration which ensures a good approximation of the real solution, (ii) the choice of an initial time instant $t_0$ and (iii) the choice of the final time instant $T=t_0+Nh$. Román-Román et al. (2008) studied the problems related to the practical application of the numerical procedure. The first problem is linked with a suitable choice of *h*. Indeed, taking into account the result of Theorem 4 of Buonocore et al. (1987), it is easy to note that the convergence is ensured when $h\rightarrow 0^+$. Consequently, the value of *h* should be small enough, but sufficiently far from 0. Indeed, if *h* is excessively small, then the computational cost may increase in vain because, with a larger integration step, a similar approximation may be obtained with a smaller number of iterations. On the other hand, if *h* is excessively large, the approximation may be unsatisfactory. These problems depend on the localization of the FPT *T*, and may be solved if the range of variation of *T* is known. For this reason, Román-Román et al. (2008) introduced a function, called ‘FPT location’ (FPTL), finalized to obtain, from a heuristic point of view, the range of variation of *T*. Specifically, the FPTL function is defined as follows

$$\begin{aligned} \begin{aligned} FPTL(t)&={\left\{ \begin{array}{ll} {\mathbb {P}}\left[ X(t)>S(t)|X(t_0)=x_0\right] ,\quad x_0<S(t_0)\\ {\mathbb {P}}\left[ X(t)<S(t)|X(t_0)=x_0\right] ,\quad x_0>S(t_0) \end{array}\right. }\\&={\left\{ \begin{array}{ll} 1-F\left( S(t),t|x_0,t_0\right) ,\quad x_0<S(t_0)\\ F\left( S(t),t|x_0,t_0\right) ,\quad x_0>S(t_0), \end{array}\right. } \end{aligned} \end{aligned}$$

where $F(x,t|x_0,t_0)$ is the transition distribution of the process *X*(*t*). Referring to the diffusion process with infinitesimal moments given by Eq. (7), and by considering a fixed and constant boundary $S>x_0$, given $X(t_0)=x_0$, the FPTL function for the process *X*(*t*) is given by

$$\begin{aligned} FPTL(t)=1-\varPhi \left( C(t)\right) , \end{aligned}$$

where $\varPhi$ is the standard normal distribution and

$$\begin{aligned} C(t)=\frac{1}{\sigma \sqrt{t-t_0}}\left\{ \log S-\log x_0 -\log \left[ \frac{\eta +e^{-Q_\beta (t_0)}}{\eta +e^{-Q_\beta (t)}}\right] +\frac{\sigma ^2}{2}(t-t_0)\right\} . \end{aligned}$$

The information provided by the FPTL function is relevant for an efficient application of the algorithm proposed by Buonocore et al. (1987). Indeed, thanks to the FPTL function, an adaptive step of integration can be obtained. In this way, the execution time of the algorithm is reduced.

### Example 1

**Fig. 3:**
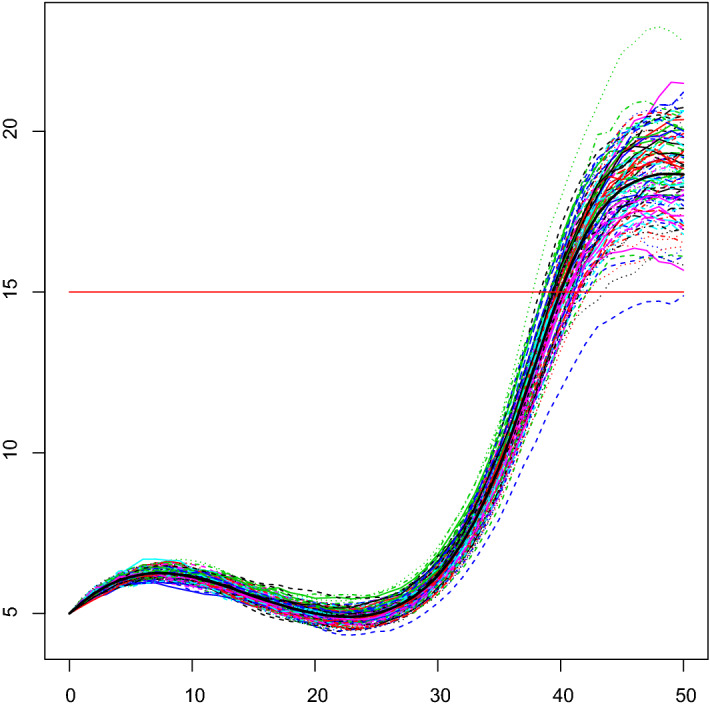
100 simulated sample paths of the process *X*(*t*) with $p=3$, $Q_\beta (t)=0.1t-0.009t^2+0.0002t^3$, $\eta =e^{-1}$, $\sigma =0.01$, $t_0=0$ and $x_0=5$. The black line represents the sample mean of the process, while the red line represents the boundary $S=15$

Let *X*(*t*) be a diffusion process with infinitesimal moments (7), with $p=3$, $Q_\beta (t)=0.1t-0.009t^2+0.0002t^3$, $\eta =e^{-1}$, $\sigma =0.01$, $t_0=0$, and $X_0=5$ a.s. See Fig. 3 for the plot of 100 simulated sample paths of the process. Let us study the FPT density through the fixed boundary $S=3\, x_0=15$, by using the information provided by the FPTL function and the R package fptdApprox (for references, see Román-Román et al. (2012), Román-Román et al. (2014) and Román-Román et al. (2020)).

**Fig. 4:**
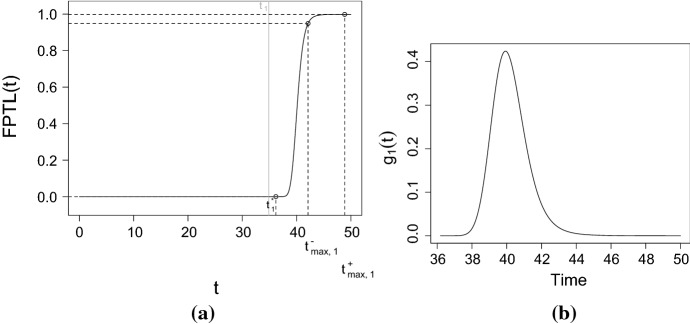
**a** The FPTL function and **b** the approximated FPT density of the process *X*(*t*) through the constant boundary $S=15$, for the same assumptions of Fig. 3

Figure 4a shows the FPTL function (obtained by means the function FPTL of the package fptdApprox), whereas the approximated FPT density (obtained using the package fptdApprox) is plotted in Fig. 4b. Other useful quantities related to the FPT density are given in Table 1.

## Simulation

**Fig. 5:**
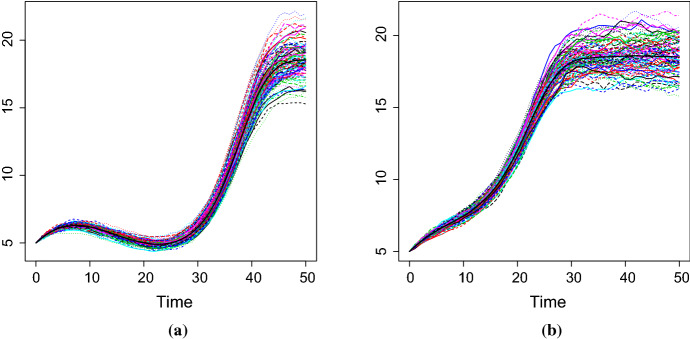
100 simulated sample paths of the diffusion process for $\sigma =0.01$, $\eta =e^{-1}$ and **a** $\beta _1=0.1$, $\beta _2=-0.009$, $\beta _3=0.0002$ and **b** $\beta _1=0.1$, $\beta _2=-0.007$, $\beta _3=0.0003$ (simulation study)

In Sect. 3, two procedures have been introduced to obtain the MLEs of the parameters involved in the diffusion process (9). The former procedure is based on the numerical resolution of a system of nonlinear equations, whereas the latter is based on the application of S.A. algorithm. In this section, a simulation study is developed to verify the validity of the two aforementioned procedures. We consider the diffusion process *X*(*t*) with infinitesimal moments (7), for $p=3$, and $\beta _1\in \left\{ 0.1,0.5\right\}$, $\beta _2\in \left\{ -0.009,-0.007\right\}$, $\beta _3\in \left\{ 0.0002, 0.0004\right\}$, $\eta \in \left\{ e^{-1},e^{-3}\right\}$ and $\sigma \in \left\{ 0.01,0.05\right\}$. These choices of the parameters are performed arbitrarily, to obtain different patterns of the growth curve. For example, the choice $\beta _1=0.1$, $\beta _2=-0.009$, $\beta _3=0.0002$, $\eta =e^{-1}$ refers to the case of a non monotonous multisigmoidal logistic function, whereas the choice $\beta _1=0.1$, $\beta _2=-0.007$, $\beta _3=0.0003$ and $\eta =e^{-1}$ to the case of an increasing multisigmoidal logistic curve (see Fig. 5). To estimate the parameters in $\xi$, we consider the 32 combinations of the values of the parameters listed in Table 2, with $x_0=5$ in every case. For each case, we simulate 200 sample paths of *X*(*t*), by generating 501 simulated points at equidistant times for $0\le t\le 50$.

**Table 1:** The mean, the standard deviation, the mode, the first, the fifth and the ninth decile of the FPT of the process *X*(*t*) through the boundary $S=15$

Mean	St. dev.	Mode	1st decile	5th decile	9th decile
40.18765	1.568392	39.92321	39.02346	40.11264	41.58065

**Table 2:** The values of the parameters (simulation study)

Case no.	$\beta _1$	$\beta _2$	$\beta _3$	$\eta$	$\sigma$	Case no.	$\beta _1$	$\beta _2$	$\beta _3$	$\eta$	$\sigma$
1	0.1	$-0.009$	0.0002	$e^{-1}$	0.01	17	0.5	$-0.009$	0.0002	$e^{-1}$	0.01
2	$''$	$''$	$''$	$''$	0.05	18	$''$	$''$	$''$	$''$	0.05
3	$''$	$''$	$''$	$e^{-3}$	0.01	19	$''$	$''$	$''$	$e^{-3}$	0.01
4	$''$	$''$	$''$	$''$	0.05	20	$''$	$''$	$''$	$''$	0.05
5	$''$	$''$	0.0003	$e^{-1}$	0.01	21	$''$	$''$	0.0003	$e^{-1}$	0.01
6	$''$	$''$	$''$	$''$	0.05	22	$''$	$''$	$''$	$''$	0.05
7	$''$	$''$	$''$	$e^{-3}$	0.01	23	$''$	$''$	$''$	$e^{-3}$	0.01
8	$''$	$''$	$''$	$''$	0.05	24	$''$	$''$	$''$	$''$	0.05
9	$''$	$-0.007$	0.0002	$e^{-1}$	0.01	25	$''$	$-0.007$	0.0002	$e^{-1}$	0.01
10	$''$	$''$	$''$	$''$	0.05	26	$''$	$''$	$''$	$''$	0.05
11	$''$	$''$	$''$	$e^{-3}$	0.01	27	$''$	$''$	$''$	$e^{-3}$	0.01
12	$''$	$''$	$''$	$''$	0.05	28	$''$	$''$	$''$	$''$	0.05
13	$''$	$''$	0.0003	$e^{-1}$	0.01	29	$''$	$''$	0.0003	$e^{-1}$	0.01
14	$''$	$''$	$''$	$''$	0.05	30	$''$	$''$	$''$	$''$	0.05
15	$''$	$''$	$''$	$e^{-3}$	0.01	31	$''$	$''$	$''$	$e^{-3}$	0.01
16	$''$	$''$	$''$	$''$	0.05	32	$''$	$''$	$''$	$''$	0.05

The remainder of this section is organized as follows: (a) since the degree of the polynomial $Q_\beta$ is unknown a priori, we propose the use of the strategy described in Román-Román et al. (2019), by increasing the degree until the goodness of fit is optimal; (b) considering the degree obtained at the step (a), we use the two procedures described in Sects. 3.1 and 3.2 to find the MLEs of the parameters.

The choice of the best degree of the polynomial $Q_\beta$ is performed under the goodness of fit criteria based on the four following measures:

(i) the absolute relative error (*RAE*) between the sample mean and the estimated mean, i.e.
  $$\begin{aligned} RAE_p=\frac{1}{N}\sum _{i=1}^N\frac{\left| m_i-\hat{\mathbb E}(X^{(p)}(t_i))\right| }{m_i},\qquad p=2,3,\dots , \end{aligned}$$
  where $\hat{{\mathbb {E}}}(X^{(p)}(t_i))$ denotes the mean of the estimated process considering a polynomial $Q_\beta$ of degree *p*;
(ii) the Akaike information criterion (*AIC*), which is defined as
  $$\begin{aligned} AIC_p=2(p+2)-2L_{{\mathbb {V}}}({{\hat{\alpha }}}, {{\hat{\xi }}}), \qquad p=2,3\dots , \end{aligned}$$
(iii) the Bayesian information criterion (*BIC*), which is given by
  $$\begin{aligned} BIC_p=(p+2)\log (n)-2L_{{\mathbb {V}}}({{\hat{\alpha }}}, {{\hat{\xi }}}), \qquad p=2,3\dots , \end{aligned}$$
  where *n* represents the number of observations,
(iv) the resistor-average distance ($D_{RA}$) between the sample distribution $f_C$ and the *p*-th estimated distribution $f_{S_p}$, for $p=2,\dots ,6$, which is defined as the following harmonic mean (cf. Johnson and Sinanovic (2001)):
  $$\begin{aligned} D_{RA}(f_C||f_{S_p})(t)=\frac{D_{KL}(f_C||f_{S_p}) (t)\cdot D_{KL}(f_{S_p}||f_{C}) (t)}{D_{KL}(f_C||f_{S_p}) (t) + D_{KL}(f_{S_p}||f_{C}) (t)}, \qquad t\ge t_0, \end{aligned}$$
  where $D_{KL}$ denotes the Kullback-Leibler divergence. Assuming that the sample distribution is lognormal with parameters
  $$\begin{aligned} \mu _C(t)\approx {\widehat{\mu }}_t= \log (m_g(t)), \qquad \sigma _C^2(t)\approx {\widehat{\sigma }}_t^2=2\log \frac{m(t)}{m_g(t)}, \end{aligned}$$
  and that the estimated distribution is lognormal with parameters
  $$\begin{aligned} \mu (t)\approx {\widehat{\mu }}_0+H_{{{\widehat{\xi }}}}(t_0,t), \qquad \sigma (t)\approx {\widehat{\sigma }}_0^2+{\widehat{\sigma }}^2(t-t_0), \end{aligned}$$
  the Kullback-Leibler divergence between the sample distribution $f_C$ and the *p*-th estimated distribution $f_{S_p}$ for $p=2,\dots ,6$ is given by, for any $t\ge t_0$
  $$\begin{aligned}&D_{KL}(f_C||f_{S_p})(t)\\&\qquad =\frac{1}{2}\left[ \log \left( \frac{{\widehat{\sigma }}_0^2+{{\widehat{\sigma }}}^2(t-t_0)}{{\widehat{\sigma }}_t^2}\right) +\frac{{\widehat{\sigma }}_t^2+\left( {{\widehat{\mu }}}_t-{\widehat{\mu }}_0-H_{{{\widehat{\xi }}}}(t_0,t)\right) ^2}{{\widehat{\sigma }}_0^2+{{\widehat{\sigma }}}^2(t-t_0)}-1\right] , \end{aligned}$$
  with $H_{{{\widehat{\xi }}}}(t_0,t)$ defined in (10). Clearly, if the theoretical distribution of the process is known, one can alternatively compute the resistor-average distance between the theoretical and the estimated distribution. We consider the expected distance and the median of the distance as reference values for the resistor-average distance.

**Table 3:** The estimated parameters obtained by solving system (23) for different degrees of the polynomial $Q_\beta$ (simulation study)

	Value	$\beta _1$	$\beta _2$	$\beta _3$	$\beta _4$
$p=2$	Initial	$-0.19227458$	0.006134021	–	–
	Estimated	$-0.02167632$	0.001417851	–	–
$p=3$	Initial	$-0.02332665$	$-0.005246427$	0.0001724780	–
	Estimated	0.10200076	$-0.009115730$	0.0002019617	–
$p=4$	Initial	$-0.16488586$	0.011922169	$-0.0004348227$	$6.54664\mathrm{{e}}{-}03$
	Estimated	0.10313862	$-0.009310721$	0.0002108884	$-1.240487\mathrm{{e}}{-}07$
$p=5$	Initial	$-0.23209031$	0.024605250	$-0.0012041705$	$2.521331\mathrm{{e}}{-}05$
	Estimated	$-0.23209031$	0.024605250	$-0.0012041705$	$2.521331\mathrm{{e}}{-}05$
$p=6$	Initial	$-0.36570406$	0.060641889	$-0.0044843917$	$1.579124\mathrm{{e}}{-}04$
	Estimated	$-0.36570406$	0.060641889	$-0.0044843917$	$1.579124\mathrm{{e}}{-}04$
$p=2$	Initial	–	–	0.3688831	0.0092356136
	Estimated	–	–	0.2342132	0.0013732663
$p=3$	Initial	–	–	0.3688831	0.0092356136
	Estimated	–	–	0.3649943	0.000103298
$p=4$	Initial	–	–	0.3688831	0.0092356136
	Estimated	–	–	0.3649325	0.0001032229
$p=5$	Initial	$-1.572548\mathrm{{e}}{-}07$	–	0.3688831	0.0092356136
	estimated	$-1.572548\mathrm{{e}}{-}07$	–	0.3688831	0.0000859656
$p=6$	Initial	$-2.617632\mathrm{{e}}{-}06$	$1.706174\mathrm{{e}}{-}08$	0.3688831	0.0092356136
	Estimated	$-2.617632\mathrm{{e}}{-}06$	$1.706174\mathrm{{e}}{-}08$	0.3688831	0.0000859656

**Table 4:** The goodness measures for different degree

Degree	*RAE*	*BIC*	*AIC*	Median of $D_{RA}$	Mean of $D_{RA}$
2	0.163198755	$-$6986.46	$-7006.983$	1.076721237	1.874847040
3	0.001667815	$-$10261.07	$-10286.721$	0.001035228	0.001668560
4	0.001390662	$-$10254.13	$-10284.916$	0.001149273	0.001916131
5	0.413978453	34449.77	34449.77	54.690728902	84.305882377
6	0.416847669	47306.94	47306.94	69.316641926	94.836470468

For the resistor-average distance $D_{RA}$, the estimated and the theoretical distributions are considered (simulation study)

**Fig. 6:**
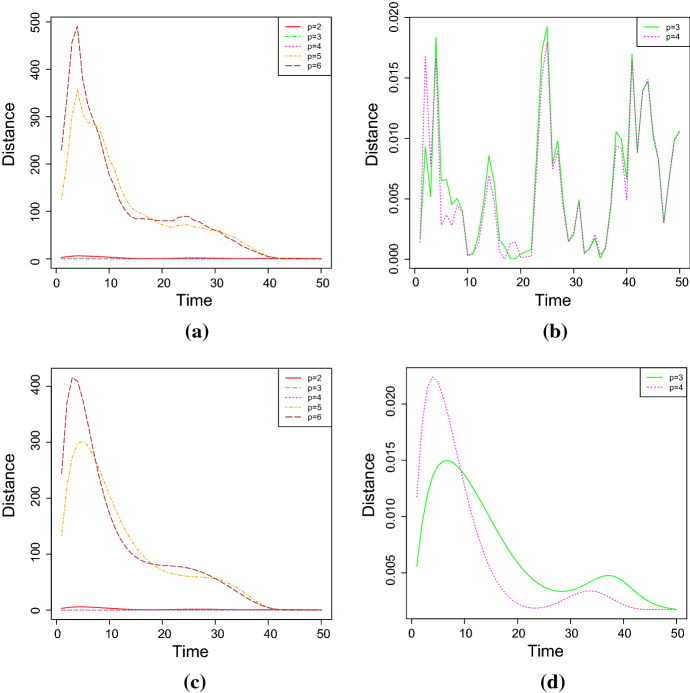
The resistor average distance between **a**–**b** the sample and the estimated distribution, and **c**–**d** the theoretical and estimated distribution for the case 1 of Table 2, for different degrees of the polynomial (simulation study)

**Table 5:** The estimated values of the parameters obtained by solving the system (simulation study)

Case no.	${\hat{\beta }}_1$	${\hat{\beta }}_2$	${\hat{\beta }}_3$	${{\hat{\eta }}}$	${{\hat{\sigma }}}$	*RAE*
1	0.1008708	$-0.009083141$	0.0002016053	0.3748345	0.009948509	0.009948509
2	0.09647959	$-0.009128214$	0.0002035713	0.4174093	0.04861361	0.00968444
3	0.1006515	$$-0.009032199 $$	0.0002004328	0.04941139	0.01006651	0.001394514
4	0.09813563	$-0.008927315$	0.0001989717	0.05311648	0.05045509	0.004833166
5	0.09917466	$-0.008943618$	0.0002994268	0.3684543	0.009948515	0.0007958334
6	0.1014043	$$-0.008998974 $$	0.0002981807	0.3892023	0.05016611	0.004735055
7	0.1000035	$-0.008977959$	0.000299122	0.0499255	0.01019311	0.001671998
8	0.0998637	$-0.009123996$	0.0003010265	0.05329735	0.04990423	0.006741047
9	0.0996273	$$-0.006921284 $$	0.000198009	0.3691476	0.01007512	0.001185446
10	0.09453137	$-0.006522038$	0.0001887209	0.3961056	0.05014104	0.008249555
11	0.0994893	$-0.006961563$	0.000199408	0.05006629	0.01005502	0.001227369
12	0.09991228	$-0.007300655$	0.0002073627	0.05440441	0.04977635	0.008537779
13	0.09871006	$-0.006934799$	0.0002995149	0.3732344	0.009929474	0.001391576
14	0.1089433	$-0.00769021$	0.0003170773	0.3827415	0.05020807	0.007503426
15	0.09915895	$-0.006970525$	0.0003002155	0.05005078	0.009887142	0.001247189
16	0.099621	$-0.007308948$	0.0003089962	0.05292541	0.05039523	0.01051539
17	0.5027283	$-0.009975521$	0.0002718814	0.3675535	0.01005231	0.001725124
18	0.4994427	$-0.0113279$	0.0008357429	0.3763182	0.04958661	0.01836523
19	0.5009121	$-0.009399375$	0.0002318097	0.04989883	0.01014064	0.001231152
20	0.4889807	$-0.007392059$	0.0001686769	0.05133072	0.05002574	0.02180217
21	0.4966796	$-0.005064475$	$9.070535\mathrm{{e}}{-}05$	0.3645112	0.008760448	0.00882512
22	0.5169986	$-0.008982219$	0.0007673324	0.3842942	0.05027921	0.01926412
23	0.5007486	$-0.00918498$	0.0003122469	0.05015442	0.009886917	0.001718233
24	0.5004829	$-0.009757746$	0.0004111398	0.05113107	0.0501877	0.03498688
25	0.4992278	$-0.007530001$	0.0002736762	0.367121	0.01017701	0.003303379
26	0.522767	$-0.01616745$	0.001661617	0.3978223	0.0504873	0.02065117
27	0.4947951	$-0.004930811$	$-3.751225\mathrm{{e}}{-}05$	0.04872577	0.0100036	0.01431122
28	0.4973394	$-0.004424543$	$-5.398909\mathrm{{e}}{-}05$	0.0509033	0.04989332	0.008392656
29	0.5007914	$-0.005683407$	0.0001988298	0.3686014	0.01002725	0.001054943
30	0.6589188	$-0.01472611$	$7.767629\mathrm{{e}}{-}05$	0.3884501	0.05398134	0.02949292
31	0.5005393	$-0.007046342$	0.0003130846	0.04990925	0.009948212	0.002032919
32	0.4944903	$-0.006618593$	0.0004977852	0.0531368	0.05019473	0.01046167

In cases (ii) and (iii), the stochastic model is characterized by $p+2$ parameters. Moreover, $L_{\mathbb {V}}(\alpha ,\xi )$ is defined in (17), and ${{\hat{\alpha }}}$ and ${{\hat{\xi }}}$ are the MLEs of the parameters $\alpha$ and $\xi$. The best fit is attained for the smallest value of the considered goodness measures. Table 3 shows the estimated parameters for the case no. 1 of Table 2, which is obtained by solving the system (23) for different degrees of the polynomial $Q_\beta$. Furthermore, the results about the goodness of measures are given in Table 4 and in Fig. 6. It can be noticed that the estimated parameters for $p=3$ and $p=4$ are almost identical, and that $\beta _4$ is very close to zero in the case $p=4$. Hence, the results concerning the measures of goodness obtained in these two cases are quite similar. This conclusion is also confirmed by the analysis of the RAE measures (in Table 4), that are often used to measure the fit error of the model in terms of the fit of the mean function. The analysis is performed in terms of the scale of judgment of the model accuracy based on the Mean Absolute Percentage Error (MAPE), cf. Klimberg et al. (2010) and Lewis (1982). Indeed, the judgment suggested by the MAPE shows that $p=3$ and $p=4$ are referred as highly accurate, whereas $p=2$ is evaluated as good forecast, with both $p=5$ and $p=6$ considered as reasonable forecast. Consequently, the choice $p=3$ is taken as the best, since it involves the lowest number of parameters.

**Fig. 7:**
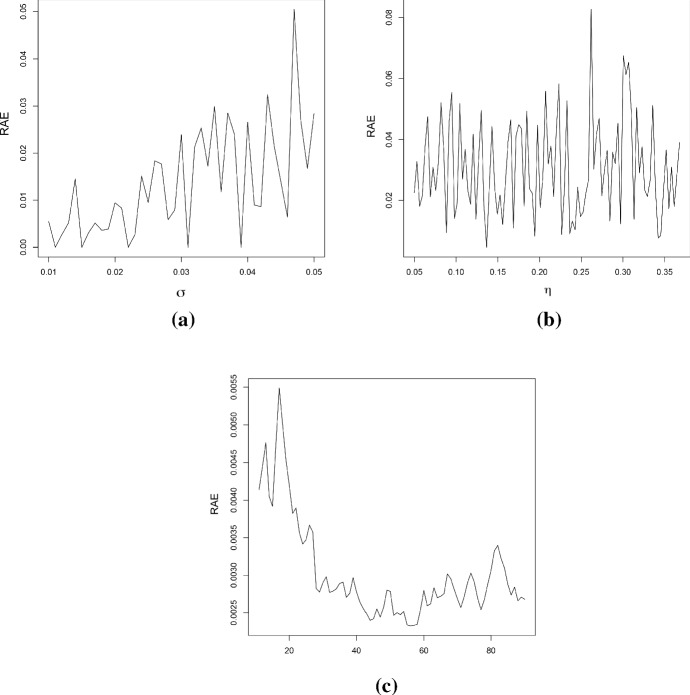
The *RAE* for **a** $\eta =e^{-1}$ and $\sigma \in [0.01,0.05]$, **b** $\sigma =0.05$ and $\eta \in \left[ e^{-3},e^{-1}\right]$ and **c** $\sigma =0.01$, $\eta =e^{-1}$ and with respect of the number of replications. In all the cases $Q_\beta (t)=0.1t-0.009t^2+0.0002t^3$ (simulation study)

**Fig. 8:**
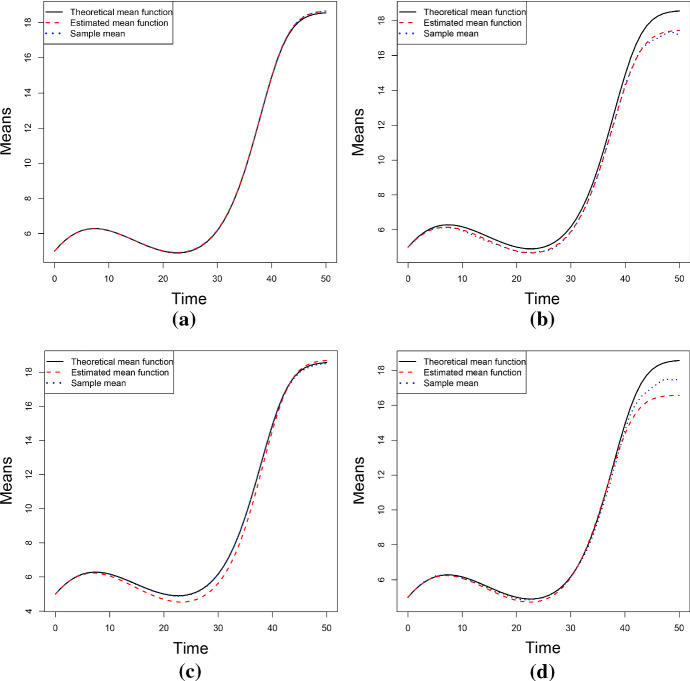
The theoretical, sample and estimated means of the process *X*(*t*) for the parameters of the cases number 1 and 2 of Table 2 (from left to right). The results are obtained via Newton-Raphson method in (**a**) and (**b**) and via S.A. in (**c**) and (**d**). (Simulation study)

The same result can be obtained for the other parameters choices, but it is omitted for brevity. Here, we limit to mention that the AIC and its Bayesian version, the BIC, provide a global measure of the adjustment to the model in terms of the likelihood that the model itself gives to the observed sample, so that these measures also allow for model selection criteria. The AIC and the BIC are seen often as complementary measures to the use of the Resistor Average Distance between the sample and the theoretical distributions of the model. However, there is no criterion that indicates that one measure is better than another, and thus in general the use of several alternative measures is recommended, as usual in practical applications. In our analysis, the coincidence of the conclusions suggested by these measures supports the final decision. Hence, from now on, a polynomial of degree $p=3$ will be considered.

Table 5 shows the estimated values of the parameters obtained by solving the nonlinear system (23) by means of the Newton-Raphson method. These values provide good parameters estimates, especially when $\sigma$ is small. The last column of the Table 5 contains the *RAE*. In this case, it is defined as

28 $$\begin{aligned} RAE_3=\frac{1}{N}\sum _{i=1}^N\frac{\left| m_i-\hat{\mathbb E}(X^{(3)}(t_i))\right| }{m_i}, \end{aligned}$$

where $m_i$ are the values of the sample mean and $\hat{\mathbb E}(X^{(3)}(t_i))$ are the values of the estimated mean at the time $t_i$ considering a polynomial of degree $p=3$. For a comparison between $\sigma$ or $\eta$ and the *RAE*, see Fig. 7a–b: it can be noticed that the value of the *RAE* shows an increasing trend with respect to the parameter $\sigma$, whereas it shows a constant trend with respect to $\eta$. In Fig. 8a–b the theoretical, sample and estimated sample means for the parameters choices number 1 and 2 of the Table 2 are shown. Clearly, the best estimation is obtained when $\sigma$ is small.

Further on, the estimated values obtained via S.A. are given in Table 6, whose last column contains the value of the *RAE* defined in Eq. (28). Since S.A. is a heuristic algorithm, the *MLEs* have been computed as the average of the results obtained by 10 uses of the procedure. Figure 8c–d provide the theoretical, the sample and the estimated (via S.A.) means for the cases no. 1 and no. 2 of the Table 2. In addition, in Fig. 7c, the trend of the *RAE* is plotted as a function of the number of replications: clearly, the goodness of the results improves as the number of replications increases.

Moreover, Table 7 contains the estimated values of the parameters (obtained by solving the system (23)), as well as their real values and the asymptotic estimation error. Finally, Table 7 provides various confidence intervals obtained by applying the delta method and using the distribution given in Sect. 3.3 for the case no. 1 of Table 2.

### Approximation of FPT density

**Fig. 9:**
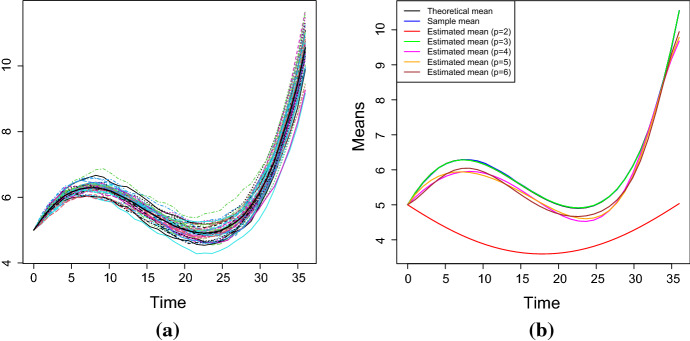
**a** 50 simulated sample paths of the diffusion process *X*(*t*) for $\sigma =0.01$ , $\eta =e^{-1}$ and $Q_\beta (t)=0.1t-0.009t^2+0.0002t^3$. **b** Theoretical, sample and estimated means of the process *X*(*t*) for the FPT density approximation (simulation study—FPT problem)

In this section, the FPT problem is analyzed. With reference to a diffusion process *X*(*t*) with a multisigmoidal logistic mean and $Q_\beta (t)= 0.1t-0.009t^2+0.0002t^3$, $\eta =e^{-1}$ and $\sigma =0.01$, we construct 50 simulated sample paths (see Fig. 9a), each one being formed by 361 data simulating $X(t_i)$ for $t_i=(i-1)\, 0.1$, $i=1,\dots , 361$. As in Sect. 5, we first chose the optimal polynomial degree (which corresponds to the best fit), and then we found the MLEs of the parameters by solving the system (23). Further on, the R package fptdApprox is used to approximate the FPT density of the process through a constant threshold $S=15$. Table 8 provides the estimated parameters, whereas Fig. 9b shows the theoretical, the sample and the estimated means, for $p=2,3,4,5,6$.

**Table 6:** The estimated values of the parameters obtained via S.A. (simulation study)

Case no.	${\hat{\beta }}_1$	${\hat{\beta }}_2$	${\hat{\beta }}_3$	${{\hat{\eta }}}$	${\hat{\sigma }}^2$	*RAE*
1	0.101561	$-0.009082552$	0.0002018522	0.3625206	$9.989558\mathrm{{e}}{-}05$	0.01145807
2	0.09954966	$-0.009144748$	0.0002033654	0.4041588	0.002549043	0.04471631
3	0.1131058	$-0.00993448$	0.0002154949	0.05040806	0.0001547924	0.02141325
4	0.1091467	$-0.009814184$	0.0002144671	0.05347671	0.002515041	0.0371569
5	0.1180621	$-0.01109313$	0.0003614234	0.3760972	0.0001248723	0.01352716
6	0.1278859	$-0.01283086$	0.0004142981	0.4122873	0.00251747	0.01633801
7	0.1060342	$-0.009544155$	0.00031209	0.0501014	0.0001160831	0.007163973
8	0.1233499	$-0.01139476$	0.000355587	0.05196034	0.002510716	0.02342116
9	0.05999024	$$-0.003213622 $$	0.0001156288	0.3619285	0.00019005	0.02598964
10	0.1281187	$-0.01053716$	0.000303384	0.4643666	0.002569706	0.04634721
11	0.07833233	$-0.005157051$	0.0001608136	0.05258961	0.0001810726	0.04867231
12	0.118225	$-0.008582805$	0.0002342187	0.05132632	0.002638776	0.035553561
13	0.1177451	$-0.00947922$	0.0003863382	0.3979672	0.0002070029	0.02261541
14	0.1155341	$-0.009715339$	0.0004058845	0.4262232	0.002535962	0.01010711
15	0.09977635	$-0.007003481$	0.0003003812	0.04986716	0.0001068239	0.001787891
16	0.1240548	$-0.009570475$	0.0003701909	0.05082801	0.002543112	0.02769778
17	0.4996174	$-0.007431545$	$1.808038\mathrm{{e}}{-}05$	0.3697584	$9.898729\mathrm{{e}}{-}05$	0.003974179
18	0.5116561	$-0.01727471$	0.0002780168	0.3700344	0.002477853	0.01019564
19	0.4908564	$-0.007392949$	$5.594539\mathrm{{e}}{-}05$	0.04968137	0.0001481544	0.008720396
20	0.5013114	$-0.00809688$	0.0001788676	0.0530825	0.00273835	0.009876328
21	0.6249235	$-0.02166049$	0.000398154	0.4112333	0.000224436	0.06819835
22	0.5686069	$-0.02374947$	0.000553332	0.3847588	0.002566421	0.01345391
23	0.4865293	$-0.006591622$	$5.45598\mathrm{{e}}{-}05$	0.05038445	0.0001598932	0.01251157
24	0.4833729	$-0.00633273$	0.000290948	0.05788054	0.002589166	0.03791299
25	0.4994063	$-0.008634032$	$9.26605\mathrm{{e}}{-}05$	0.3633383	0.0001204934	0.003431125
26	0.4987191	$-0.004809727$	$6.356344\mathrm{{e}}{-}05$	0.3925613	0.002584155	0.02507006
27	0.5200495	$-0.01434754$	0.001020145	0.05181291	0.0001540365	0.02603051
28	0.4965627	$-0.004536852$	0.0001049973	0.05176822	0.002566167	0.01328192
29	0.4859707	$-0.005580455$	$9.861833\mathrm{{e}}{-}05$	0.3638371	0.0001267092	0.006797028
31	0.4917976	$-0.004818751$	0.0001038265	0.04923269	0.0001633201	0.01411135
32	0.5002401	$-0.008964678$	0.0006969377	0.05423012	0.002528847	0.02389477

**Table 7:** The estimated values of the parameters (obtained by solving the system (23)), their real values, their asymptotic estimation error and their $95\%$, $90\%$ and $75\%$ confidence intervals (simulation study)

Parametric function	$\eta$	$\beta _1$	$\beta _2$
Estimated value	0.3748345	0.1008708	$-0.0090831$
Real value	0.3678794	0.1000000	$-0.0090000$
Standard error	$1.218018\mathrm{{e}}{-}03$	$5.355428\mathrm{{e}}{-}05$	$2.683426\mathrm{{e}}{-}07$
$95\%$ confidence interval	(0.3064315, 0.4432375)	(0.0865276, 0.1152140)	$(-0.0100984,-0.0099352)$
$90\%$ confidence interval	(0.3174289, 0.4322401)	(0.0888336, 0.1129080)	$(-0.0099352,-0.0082311)$
$75\%$ confidence interval	(0.3346872, 0.4149818)	(0.0925245, 0.1092891)	$(-0.0096790, -0.0084872)$

Parametric function	$\beta _3$	$\sigma ^2$	
Estimated value	0.0002016	0.0000990	
Real value	0.0002000	0.0001000	
Standard error	$9.218516\mathrm{{e}}{-}11$	$3.918248\mathrm{{e}}{-}12$	
$95\%$ confidence interval	(0.0001828, 0.0002204)	(0.0000951, 0.0001028)	
$90\%$ confidence interval	(0.0001858, 0.0002174)	(0.0000957, 0.0001022)	
$75\%$ confidence interval	(0.0001906, 0.0002126)	(0.0000967, 0.0001012)	

**Fig. 10:**
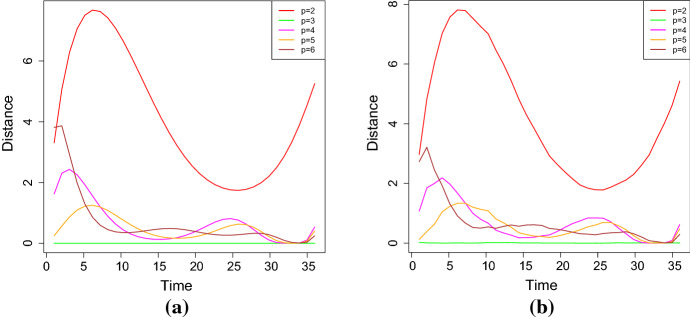
Resistor-average distance between **a** the theoretical and the estimated distributions and **b** between the sample and the estimated distributions for the FPT density approximation (simulation study - FPT problem)

Table 9 provides the four goodness measures for the considered degrees *p* of the polynomial $Q_\beta$. Figure 10 shows the resistor-average distances between the theoretical and the estimated distributions, and also between the sample and the estimated distributions. From the given results it follows that the best degree is $p=3$.

**Table 8:** The estimated values of the parameters considering $p=2,3,4,5,6$ for the FPT density approximation (simulation study - FPT problem)

Degree	$\eta$	$\beta _1$	$\beta _2$	$\beta _3$
$p=2$	2.5287214	$-0.09696011$	0.002712063	–
$p=3$	0.3425411	0.09735434	$-0.008730983$	0.0001936518
$p=4$	0.9005909	0.08465459	$-0.003953699$	$-0.0001821386$
$p=5$	0.9005909	0.12376611	$-0.014259773$	0.0006907176
$p=6$	0.9005909	0.04089375	0.016958673	$-0.0032783145$

Degree	$\beta _4$	$\beta _5$	$\beta _6$	$\sigma ^2$
$p=2$	–	–	–	0.0017740001
$p=3$	–	–	–	$1.025400\mathrm{{e}}{-}04$
$p=4$	$7.788128\mathrm{{e}}{-}06$	–	–	$8.511030\mathrm{{e}}{-}05$
$p=5$	$-2.178008\mathrm{{e}}{-}05$	$3.477652\mathrm{{e}}{-}07$	–	$8.511030\mathrm{{e}}{-}05$
$p=6$	$2.024839\mathrm{{e}}{-}04$	$-5.459791\mathrm{{e}}{-}06$	$5.624755\mathrm{{e}}{-}08$	$8.511030\mathrm{{e}}{-}05$

**Table 9:** The *RAE*, the *AIC*, the *BIC* the median and the mean of the resistor-average distance $D_{RA}$ of the parameters for $p=2,3,4,5,6$

Measure of goodness	$p=2$	$p=3$	$p=4$	$p=5$	$p=6$
*RAE*	0.313009817	0.001985486	0.046745173	0.044842133	0.046064145
*AIC*	$-9288.882$	$-14314.158$	$-10687.536$	$-11569.711$	$-12001.874$
*BIC*	$-9267.012$	$-14286.821$	$-10654.731$	$-11531.439$	$-11958.135$
Median of $D_{RA}$	3.767780356	0.003568808	0.40969122	0.347181473	0.404933011
Mean of $D_{RA}$	4.276620795	0.009886785	0.617753008	0.442544796	0.582836790

For the resistor-average distance, the estimated and the theoretical distributions are considered (simulation study—FPT problem)

**Fig. 11:**
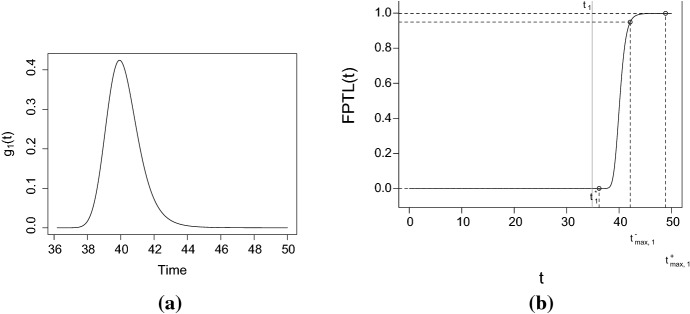
The approximated FPT density and the FPTL function of the process *X*(*t*) through the boundary $S=15$ (simulation study—FPT problem)

Using the estimated model obtained so far, we now focus on the approximation of the FPT density through the boundary $S=15$. Figure 11 shows the approximated FPT density and the FPTL function realized with the package fptdApprox. Finally, in Table 10 other useful quantities related to the FPT density are provided. It is worth noting that the results obtained in this section are in agreement with those given in Example 4.1.

**Table 10:** The mean, the standard deviation, the mode, the first, the fifth and the ninth decile of the FPT of the process *X*(*t*) through the boundary $S=15$ (simulation study—FPT problem)

Mean	St. dev.	Mode	1st decile	5th decile	9th decile
39.88883	1.034443	39.70804	38.83282	39.83612	41.0529

## Application to real data

**Fig. 12:**
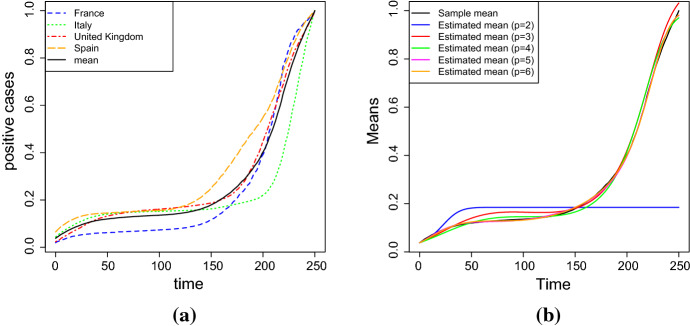
**a** Number of infections in France, Italy, Spain and United Kingdom, the black line represents the sample mean. **b** Sample and the estimated means obtained by solving the system (23) (real application)

Multisigmoidal functions are suitable to model several special growth phenomena in which the carrying capacity is reached after various stages. In any of these stages a linear growth trend is followed by an explosion of exponential type which finally flattens to a specific value. A growth of this kind is typical of some fruit species, such as peaches or coffee berries (see, for instance the application given in Sect. 3 of Di Crescenzo et al. (2021)). But also some population diseases follow an expansion with a multisigmoidal trend. In this section we apply the considered stochastic model to data concerning the COVID-19 infections in four different European countries, taken from Worldometers (2020). This is just an example finalized to show an application of the multisigmoidal logistic model, without taking into account specific more sophisticated models that describe epidemiological phenomena with greater precision. First of all, we note that the trend of infections in France, Italy, Spain and United Kingdom is similar (see Fig. 12a).

This suggests to view these data as different trajectories of the diffusion process *X*(*t*) defined on $I=[t_0,t_f]$, having a multisigmoidal logistic mean (cf. Sect. 2.1). Hence, in order to find the MLEs of the parameters, we apply the procedure described in Sect. 3.1. For each country, the initial time $t_0=0$ corresponds to the 30-th day after the one in which the number of infections exceeded 100 (March 30th for France, March 24th for Italy, March 21st for Spain, April 5th for UK), and the final time is chosen as $t_f=250$. For any path, the data are scaled as divided by their maximum value, so to be interpreted as a percentage of the last and therefore the maximum value of the growth curve. The estimated means obtained for different degrees are plotted in Fig. 12b. Table 11 provides the initial and the estimated values of the parameters, whereas Table 12 shows the four measures of goodness, for different degrees of the polynomial. Regarding the *RAE*, every time the degree increases, the approximation improves, whereas the *AIC*, the *BIC* and resistor-average distance show that the best choice is $p=3$.

**Table 11:** The initial and the estimated values of the parameters considering different degrees

	Value	$\beta _1$	$\beta _2$	$\beta _3$	$\beta _4$
$p=2$	Initial	0.00644346	$5.375995\mathrm{{e}}{-}05$	–	–
	Estimated	0.05356653	0.0011042774	–	–
$p=3$	Initial	0.04480694	$-4.587843\mathrm{{e}}{-}04$	$1.540731\mathrm{{e}}{-}06$	–
	Estimated	0.04774851	$-0.0004685118$	$1.506227\mathrm{{e}}{-}06$	–
$p=4$	Initial	0.04090832	$-3.650280\mathrm{{e}}{-}4$	$8.831071\mathrm{{e}}{-}07$	$1.405770\mathrm{{e}}{-}09$
	Estimated	0.04090832	$-0.0003650280$	$8.831071\mathrm{{e}}{-}07$	$1.405770\mathrm{{e}}{-}09$
$p=5$	Initial	0.06509648	$-1.269895\mathrm{{e}}{-}03$	$1.176365\mathrm{{e}}{-}05$	$-5.092724\mathrm{{e}}{-}08$
	Estimated	0.06509648	$-0.0012698948$	$1.176365\mathrm{{e}}{-}05$	$-5.092724\mathrm{{e}}{-}08$
$p=6$	Initial	0.05891632	$-9.396086\mathrm{{e}}{-}04$	$5.806280\mathrm{{e}}{-}06$	$-3.170420\mathrm{{e}}{-}09$
	Estimated	0.05891632	$-0.0009396086$	$5.806280\mathrm{{e}}{-}06$	$-3.170420\mathrm{{e}}{-}09$

	Value	$\beta _5$	$\beta _6$	$\eta$	$\sigma ^2$
$p=2$	Initial	–	–	0.03942583	$1.931962\mathrm{{e}}{-}02$
	Estimated	–	–	0.25862727	$2.454394\mathrm{{e}}{-}04$
$p=3$	Initial	–	–	0.03942583	$1.931962\mathrm{{e}}{-}02$
	Estimated	–	–	0.03605835	$1.024422\mathrm{{e}}{-}04$
$p=4$	Initial	–	–	0.03942583	$1.931962\mathrm{{e}}{-}02$
	Estimated	–	–	0.03942583	$3.729000\mathrm{{e}}{-}04$
$p=5$	Initial	$8.739928\mathrm{{e}}{-}11$	–	0.03942583	$1.931962\mathrm{{e}}{-}02$
	Estimated	$8.739928\mathrm{{e}}{-}11$	–	0.03942583	$3.72900\mathrm{{e}}{-}04$
$p=6$	Initial	$-8.806843\mathrm{{e}}{-}11$	$2.411350\mathrm{{e}}{-}13$	0.03942583	$1.931962\mathrm{{e}}{-}02$
	Estimated	$-8.806843\mathrm{{e}}{-}11$	$2.411350\mathrm{{e}}{-}13$	0.03942583	$3.72900\mathrm{{e}}{-}04$

The results have been obtained by solving the system (real application)

**Table 12:** *RAE*, *AIC*, *BIC*, the median and the mean of the resistor-average distance $D_{RA}$ considering different degrees

*p*	2	3	4	5	6
*RAE*	0.39085660	0.10404805	0.06853298	0.02087067	0.02030226
*AIC*	$-7225.192$	$-8175.819$	$-7582.180$	$-7677.528$	$-7664.849$
*BIC*	$$ -7205.561$$	$$-8151.281 $$	$$-7552.734 $$	$$-7643.173$$	$$-7625.587$$
Median of $D_{RA}$	0.2250753	0.1301707	0.1548300	0.1615877	0.1642668
Mean of $D_{RA}$	1.0586904	0.2741844	0.3218632	0.3103206	0.3106608

For the resistor-average distance, the estimated and the sample distributions are considered (real application)

**Fig. 13:**
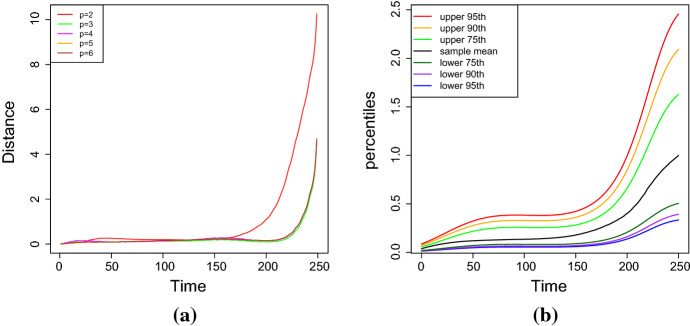
**a** The resistor-average distances between the sample and the estimated distributions considering different degrees of the polynomial. **b** The $\alpha$-percentiles of the estimated diffusion process *X*(*t*) obtained for a degree $p=3$ and for $\alpha =95,90,75$ (real application)

Regarding the last measure of goodness, see also Fig. 13a, in which the resistor-average distances between the sample and the estimated distributions are provided. Hence, in view of the results obtained for the measures of goodness, the degree $p=3$ is considered.

Table 13 shows the estimated values of the parameters, the estimation of their standard error and the $95\%$, $90\%$ and $75\%$ percentiles.

**Table 13:** The estimated values, the standard error and the $95\%$, $90\%$ and $75\%$ confidence intervals for the parameters, considering $p=3$ (real application)

Parametric function	$\eta$	$\beta _1$	$\beta _2$
Estimated value	0.03605835	0.04774851	$-0.0004685118$
Standard error	$6.086478\mathrm{{e}}{-}03$	$2.342236\mathrm{{e}}{-}03$	$2.825993\mathrm{{e}}{-}05$
$95\%$ confidence interval	(0.0241291, 0.0479876)	(0.0431578, 0.0523392)	$(-0.0005239,-0.0004131)$
$90\%$ confidence interval	(0.0260470, 0.0460697)	(0.0438959, 0.0516011)	$(-0.0005150,-0.0004220)$
$75\%$ confidence interval	(0.0290568, 0.0430599)	(0.0450541, 0.0504429)	$(-0.0005010,-0.0004360)$

Parametric function	$\beta _3$	$\sigma ^2$	
Estimated value	$1.506227\mathrm{{e}}{-}06$	$1.024422\mathrm{{e}}{-}04$	
Standard error	$9.15127\mathrm{{e}}{-}08$	$4.581353\mathrm{{e}}{-}06$	
$95\%$ confidence interval	(0.0000013, 0.0000017)	(0.0000934, 0.0001114)	
$90\%$ confidence interval	(0.0000014, 0.0000017)	(0.0000949, 0.0001010)	
$75\%$ confidence interval	(0.0000014, 0.0000016)	(0.0000971, 0.0001077)	

**Fig. 14:**
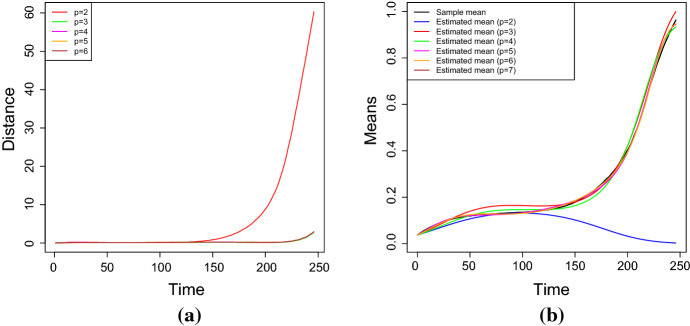
**a** Resistor-average distances between the sample and the estimated distributions in the restricted time range. **b** Sample and the estimated means obtained by solving the system (23) in the restricted time range (real application with $t_f=246$)

Moreover, the $\alpha$-percentiles (13) of the estimated diffusion process with $p=3$ are provided in Fig. 13b with $\alpha =95,90,75$. Let us now consider a restricted time range from $t_0=0$ to $t_f=246$, in order to predict the trend of the growth curve in a short-term prediction analysis. Indeed, forecasting the number of infections during a disease in progress is interesting also in the case of short terms, especially for the goodness of estimation (better in this case than in the long term analysis) and for the timeliness of the results. The considered procedure is the same of the one used above, so (i) the best degree *p* for the polynomial $Q_\beta$ is chosen by considering various measures of goodness, and (ii) the estimated values of the parameters are used to construct a diffusion process *X*(*t*) defined on $I=[0,250]$. The estimated values of the parameters are given in Table 14, the values of the four measures of goodness are given in Table 15, and finally in Fig. 14a we provide the resistor-average distances between the restricted sample and the estimated distributions. See also Fig. 14b for the plots of the estimated means for different degrees of the polynomial.

**Table 14:** The estimated values of the parameters considering $p=2,3,4,5,6$ (real application with $t_f=246$)

Degree	$\eta$	$\beta _1$	$\beta _2$	$\beta _3$
$p=2$	0.22079965	0.04165889	$-0.0002129137$	–
$p=3$	0.03650929	0.04792200	$-0.0004714958$	$1.518106\mathrm{{e}}{-}06$
$p=4$	0.04101207	0.03985192	$-0.0003277795$	$5.314482\mathrm{{e}}{-}07$
$p=5$	0.04101207	0.06778144	$-0.0013896339$	$1.350773\mathrm{{e}}{-}05$
$p=6$	0.04101207	0.05704750	$-0.0008066323$	$2.820787\mathrm{{e}}{-}06$

Degree	$\beta _4$	$\beta _5$	$\beta _6$	$\sigma ^2$
$p=2$	–	–	–	$2.536393\mathrm{{e}}{-}04$
$p=3$	–	–	–	$1.034147\mathrm{{e}}{-}04$
$p=4$	$2.392264\mathrm{{e}}{-}09$	–	–	$3.550000\mathrm{{e}}{-}04$
$p=5$	$-6.103779\mathrm{{e}}{-}08$	$1.076581\mathrm{{e}}{-}10$	–	$3.550000\mathrm{{e}}{-}04$
$p=6$	$2.602935\mathrm{{e}}{-}08$	$-2.174560\mathrm{{e}}{-}10$	$4.540655\mathrm{{e}}{-}13$	$3.550000\mathrm{{e}}{-}04$

**Table 15:** The *RAE*, the *AIC*, the *BIC*, the median and the mean of the resistor-average distance $D_{RA}$ of the parameters for $p=2,3,4,5,6$

Measure of goodness	$p=2$	$p=3$	$p=4$	$p=5$	$p=6$
*RAE*	0.41031587	0.10421156	0.07598928	0.02665995	0.02500666
*AIC*	$-2722.134$	$-8035.566$	$-7476.246$	$-7591.347$	$-7571.929$
*BIC*	$-2702.568$	$-8011.108$	$-7446.896$	$-7557.105$	$-7532.796$
Median of $D_{RA}$	0.2275183	0.1294749	0.1557215	0.1593042	0.1626228
Mean of $D_{RA}$	6.2370324	0.2212618	0.2666561	0.2532703	0.2536295

For the resistor-average distance, the estimanted and the sample distributions are considered (real application with $t_f=246$)

**Fig. 15:**
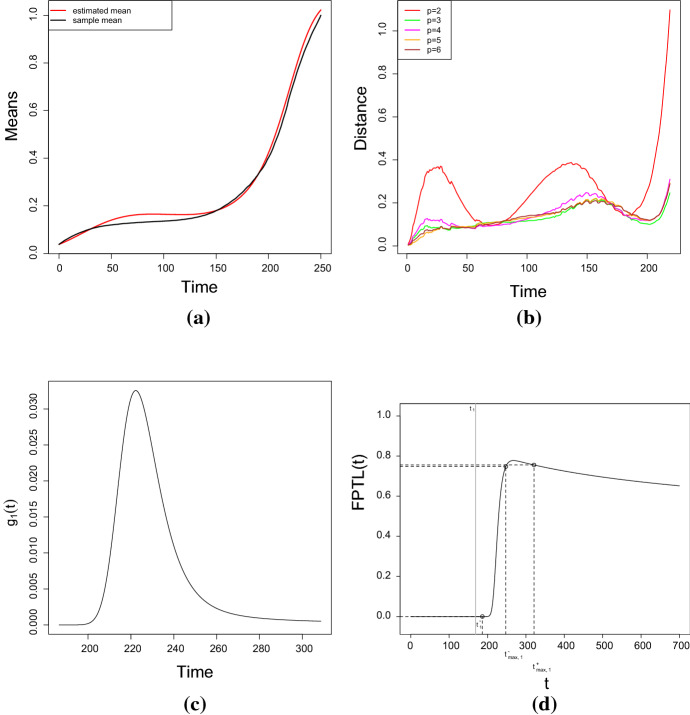
**a** The sample and the forecasted means in the complete time range $I_C=[0,250]$ considering a degree $p=3$. **b** Resistor-average distances between the sample and the estimated distributions in the restricted time range $I_R$. **c** Approximated FPT density and **d** FPTL function in the restricted time range $I_R$ through the boundary $S=0.7$ (real application—FPT problem)

Also in this case, the *RAE* does not provide a good measure of goodness, since every time the degree increases, the approximation improves. From the remaining results, the best choice is $p=3$, which corresponds to the lowest value of the *BIC* and of the *AIC*, and to the lowest resistor-average distance. Hence, considering $p=3$ and the corresponding estimated values of the parameters, Fig. 15a provides the sample and the estimated means in the complete time range, i.e. in $I_C=[0,250]$. The relative errors between the values of the sample and the estimated means are given in Table 16; note that in all cases they are less than $3\%$.

### Approximation of FPT density

This section is devoted to the FPT problem. Considering an initial portion of the available data and setting a constant boundary, an estimate of the FPT density is constructed using the numerical procedures recalled in Sect. 4. The resulting FPT density is then compared to the approximated FPT density obtained by using the whole data set.

More in detail, we consider only the first 220 data of COVID-19 infections in the restricted time range $I_R=[0, 219]$ and we investigate the best model to fit them. The choice of the optimal degree *p* of the polynomial $Q_\beta$ is based on the measures of goodness (i)–(iv) described in Sect. 5. The estimated parameters (given in Table 17) are obtained by solving the system (23).

By comparing the results given in Table 18 and in Fig. 15b, we choose $p=3$ as the optimal degree.

Then, we fix a constant threshold $S=0.7$ which corresponds to the $70\%$ of the last and maximum data in the complete time range $I_C=[0,250]$ and we use the R package fptdApprox to obtain an estimation of the FPT density. The choice of the constant boundary $S=0.7$ is not random. Indeed, it is worth observing that the descendent inflection points correspond to the peaks of the function representing the daily increments of the infections. More in detail, by means of Eq. (6), the function representing the sample mean of the infections shows two descendent inflection points, one at the time $t_{F1}=21.65$ and the other at the time $t_{F2}=220.66$. The population sizes corresponding to the inflection time instants are $S_{F1}=0.08$ and $S_{F2}=0.7$. In the time interval $[0,t_{F1}]$, the mean function has a logistic trend, hence the FPT problem through the boundary ${S_{F1}}$ is beyond the scope of the present work. Instead, since the mean in the time interval $[0, t_{F2}]$ has a multisigmoidal logistic profile, we focus our attention to the FPT problem through the threshold $S=S_{F2}=0.7$.

The approximated FPT density and the FPTL function of the estimated process *X*(*t*) through the boundary $S=0.7$ are plotted in Fig. 15c–d. In order to validate the predicted results concerning the FPT, we consider also the same problem in the complete time range $I_C$. The forecasted results for the restricted time range $I_R$ and the approximated results for the complete time range $I_C$ are given in Table 19. We note that the most meaningful index is the mode, since it corresponds to the peak of the FPT density and the two modes (namely, the mode in the restricted and in the complete time ranges) are quite close to each other (the relative error between the two values is about $1\%$).

## Conclusions

During the recent years, many sigmoidal stochastic models have been introduced to study phenomena of interest in various different scientific areas. In order to model more complex population dynamics in which the maximum level of the growth is reached after many stages, we referred to the multisigmoidal logistic stochastic growth model. More in detail, the present work has been devoted to the analysis of the corresponding statistical inference and of the FPT problem. Two procedures useful to find the MLEs of the parameters have been described, one based on the resolution of the system of the critical points of the likelihood function, and the other one based on the maximization of the likelihood function by means of the S.A. algorithm. Then, the described strategies have been validated with a simulation study. The last section of the paper has been devoted to a real application concerning COVID-19 infections in four different European countries (France, Italy, Spain and United Kingdom). The data have been fitted using a suitable multisigmoidal logistic stochastic model. Finally, a study regarding the FPT through a fixed boundary has been also performed.

**Table 16:** The sample, the forecasted mean and relative error for the last 4 time instants of the complete range $I_C=[0,250]$, considering a degree $p=3$ (real application)

Time	247	248	249	250
Sample mean	0.97179233	0.98096101	0.98949697	1.00000000
Forecasted mean	1.00554923	1.01157710	1.01720499	1.02244759
Relative error	0.03473674	0.0312103	0.02800212	0.02244759

**Table 17:** The estimated values of the parameters considering $p=2,3,4,5,6$ in the restricted time range $I_C$ (real application—FPT problem)

Degree	$\eta$	$\beta _1$	$\beta _2$	$\beta _3$
$p=2$	0.09416137	0.02514812	$-4.247417\mathrm{{e}}{-}05$	–
$p=3$	0.04858678	0.05004022	$-5.125970\mathrm{{e}}{-}04$	$1.693253\mathrm{{e}}{-}06$
$p=4$	0.06278231	0.04592707	$-4.462889\mathrm{{e}}{-}04$	$1.183743\mathrm{{e}}{-}06$
$p=5$	0.06278231	0.07198803	$-1.559548\mathrm{{e}}{-}03$	$1.646946\mathrm{{e}}{-}05$
$p=6$	0.06278231	0.05899141	$-7.664128\mathrm{{e}}{-}04$	$1.337821\mathrm{{e}}{-}07$

Degree	$\beta _4$	$\beta _5$	$\beta _6$	$\sigma ^2$
$p=2$	–	–	–	$2.63974\mathrm{{e}}{-}04$
$p=3$	–	–	–	$1.226112\mathrm{{e}}{-}04$
$p=4$	$1.721755\mathrm{{e}}{-}09$	–	–	$2.359000\mathrm{{e}}{-}04$
$p=5$	$-8.223093\mathrm{{e}}{-}08$	$1.600995\mathrm{{e}}{-}10$	–	$2.359000\mathrm{{e}}{-}04$
$p=6$	$6.730438\mathrm{{e}}{-}08$	$-4.672810\mathrm{{e}}{-}10$	$9.845081\mathrm{{e}}{-}13$	$2.359000\mathrm{{e}}{-}04$

**Table 18:** The *RAE*, the *AIC*, the *BIC*, the median and the mean of the resistor-average distance $D_{RA}$ of the parameters for $p=2,3,4,5,6$

Measure of goodness	$p=2$	$p=3$	$p=4$	$p=5$	$p=6$
*RAE*	0.28460545	0.08631377	0.06598901	0.02791701	0.03225994
*AIC*	$-6330.849$	$-7150.963$	$-6876.159$	$-6999.085$	$-6971.142$
*BIC*	$-6311.748$	$-7127.086$	$-6847.507$	$-6965.657$	$-6932.939$
Median of $D_{RA}$	0.2448219	0.1144287	0.1238069	0.1284333	0.1355944
Mean of $D_{RA}$	0.2635491	0.1260033	0.1405463	0.1325032	0.1328700

For the resistor-average distance, the estimated and the sample distributions are considered (real application—FPT problem)

**Table 19:** The mean, the mode, the 1st and the 5th deciles and the standard deviation of the FPT in the complete time range $I_C$ and in the restricted time range $I_R$ (real application—FPT problem)

Time range	Mean	Mode	1st decile	5th decile	St. deviation
Complete $I_C$	221.7308	219.8435	212.9876	221.1406	12.21116
Restricted $I_R$	197.6721	222.2305	214.8875	228.3496	80.0505

Future developments can be oriented to find the MLEs of the parameters with other meta-heuristic optimization procedures (such as Variable Neighborhood Search or other swarm-based algorithms) in order to obtain nice estimates in a short computational time. We aim also to introduce a more sophisticated model suitable to describe better epidemiological dynamics with multiple waves, starting from the multisigmoidal logistic equation. Moreover, aiming at a thorough analysis of the convergence speed for parameter estimation in stochastic differential equations, these approaches will be compared with applications of the recent method called ‘covariance matrix adaptation evolution strategy’. Indeed, the latter is used often in the presence of several parameters (cf., for instance, Ghosh et al. (2012) and Willjuice and Baskar (2010)).
